# An Improved Artificial Lemming Algorithm Integrating Non-Uniform Mutation and Q-Learning Adaptation for Underwater Manipulator Controller Tuning

**DOI:** 10.3390/biomimetics11030168

**Published:** 2026-03-02

**Authors:** Ran Wang, Weiquan Huang, Junyu Wu, Chen Chen, Yanjie Song, He Wang

**Affiliations:** 1 College of Intelligent Systems Science and Engineering, Harbin Engineering University, Harbin 150001, China; wangran407@hrbeu.edu.cn (R.W.); wujunyu@hrbeu.edu.cn (J.W.); lxcc@hrbeu.edu.cn (C.C.); wang_he@hrbeu.edu.cn (H.W.); 2 School of Information Science and Technology, Dalian Maritime University, Dalian 116026, China; songyj_2017@tju.edu.cn

**Keywords:** metaheuristic optimization, bio-inspired algorithm, improved artificial lemming algorithm, Q-learning, underwater manipulator controller, parameter tuning

## Abstract

To address the rapid population diversity loss and premature convergence of the Artificial Lemming Algorithm (ALA) in complex optimization problems, this paper proposes an Improved Artificial Lemming Algorithm (IALA) with multi-strategy enhancements inspired by lemming behavior. First, a non-uniform mutation operator and a nonlinear step-size strategy are introduced to strengthen local optima escape capability and optimization precision. Second, inspired by the foraging and positioning behavior of lemmings, a relative advantage learning strategy is designed to reduce dependence on the global best individual, further enhancing the algorithm’s exploration ability. Finally, a Q-learning-based adaptive mechanism is integrated to intelligently orchestrate five lemming-inspired behavioral modes through a nonlinear reward function, enabling adaptive switching among search patterns. Comparative experiments on the CEC2022 benchmark suite demonstrate that IALA achieves a Friedman mean rank of 1.25, ranking first with a significant margin. Compared with the original ALA and other six classical and state-of-the-art metaheuristic algorithms, and four recently proposed improved ALA variants (EALA, IALA_Tan, DMSALAs, and MsIALA), the Wilcoxon rank-sum test confirms that IALA is significantly outperformed in only 2 out of 120 pairwise comparisons, exhibiting remarkable advantages in optimization accuracy, convergence speed, and robustness. Ablation experiments validate the synergistic necessity of all three strategies, with the Q-learning adaptive mechanism identified as the most critical contributor. Exploration–exploitation balance analysis and search history visualization further confirm that IALA achieves a smooth adaptive transition from global exploration to local exploitation. Space complexity analysis reveals that the Q-table introduces only approximately 19.5 KB of fixed additional overhead, which becomes negligible for high-dimensional problems. Furthermore, IALA is successfully applied to the parameter tuning of underwater manipulator controllers, verifying its efficiency and reliability in real-world engineering applications.

## 1. Introduction

With the rapid advancement of science, technology, and engineering, real-world optimization problems have become increasingly characterized by high dimensionality, nonlinearity, multimodality, and intricate constraint structures. These complexities often render traditional deterministic optimization methods inadequate in terms of both computational efficiency and solution accuracy. It is worth noting that for optimization problems possessing exploitable mathematical structures, advanced mathematical programming approaches—such as convex relaxation techniques—can transform computationally intractable non-convex models into efficiently solvable convex formulations with global optimality guarantees [1]. However, for a broad class of real-world problems that are inherently non-convex, non-differentiable, multimodal, or black-box in nature—where the mathematical structure of the objective function is either unavailable or too complex to exploit—such exact methods become inapplicable. Meta-heuristic algorithms, which draw inspiration from biological behaviors and physical phenomena observed in nature, have emerged as powerful tools for addressing such complex optimization challenges. Their appeal stems from several key attributes: conceptual simplicity, gradient-free operation, strong robustness across diverse problem landscapes, and straightforward implementation. These nature-inspired approaches have demonstrated remarkable efficacy in a wide range of applications, including engineering design optimization [2], feature selection in machine learning [3], and autonomous path planning [4,5].

The proposal of Particle Swarm Optimization (PSO) [6] and Ant Colony Optimization (ACO) [7] laid a solid foundation for swarm intelligence algorithms and ignited a wave of research. Subsequently, to further enhance optimization performance in complex solution spaces, researchers have drawn extensive inspiration from nature, leading to the emergence of a series of high-performance intelligent algorithms. Notable examples include the Grey Wolf Optimizer (GWO) [8], Whale Optimization Algorithm (WOA) [9], Black-winged Kite Algorithm (BKA) [10], Marine Predators Algorithm (MPA) [11], Artificial Bee Colony (ABC) [12], Firefly Algorithm (FA) [13], Gannet Optimization Algorithm (GOA) [14], and the recently proposed Parrot Optimizer (PO) [15]. Beyond nature-inspired paradigms, researchers have also enriched computational intelligence by incorporating novel mathematical tools and large language model (LLM)-based technologies into algorithm design [16,17]. However, manually designing and tuning algorithm configurations for diverse problem scenarios remains laborious and expertise-intensive. Although LLM-based automatic algorithm design (AAD) offers a promising alternative, existing studies have primarily focused on traditional optimization problems and still relied on predefined algorithms with sophisticated iterative frameworks. For more intricate real-world problems—such as the EOSSP, which requires precise domain-specific articulation—the potential of LLM-driven multi-agent collaboration for fully automatic algorithm generation remains largely unexplored.

While the AAD paradigm represents a macro-level effort to automate algorithm generation, at the micro level, enhancing the performance of individual metaheuristic algorithms themselves remains equally important. The Artificial Lemming Algorithm (ALA) [18] is a novel swarm intelligence optimization algorithm introduced by Xiao et al. in 2025. Drawing inspiration from the survival behaviors of lemmings in their natural habitat, this algorithm mathematically models four distinctive behavioral patterns: long-distance migration, burrowing, foraging, and predator evasion. Compared with other metaheuristic approaches, ALA exhibits several appealing characteristics, including a well-defined algorithmic structure, minimal parameter requirements, and straightforward implementation. Nevertheless, as articulated by the “No Free Lunch” (NFL) theorem [19], no single optimization algorithm can achieve superior performance across all problem domains. When applied to complex optimization problems, the original ALA still encounters several limitations, including rapid depletion of population diversity, difficulty in achieving an effective balance between global exploration and local exploitation, and insufficient optimization precision. These deficiencies render the algorithm susceptible to premature convergence and entrapment in local optima.

To address these limitations, scholars have conducted extensive research on enhancing the performance of ALA. Tan et al. [20] proposed an improved variant that employs cooperative second-order Bernstein polynomials in conjunction with chaotic mapping strategies during the initialization phase to rectify initial position bias and enhance population diversity. During the exploitation phase, this approach constructs a smooth local approximation model through a quadratic interpolation random mutation strategy to mitigate premature convergence. Furthermore, it integrates an adaptive evolutionary strategy with lemming migration behavior, dynamically adjusting step size and direction to strengthen the capacity for escaping local optima. However, this strategy relies on predetermined rules for step size adjustment and lacks a mechanism to intelligently adapt behavioral modes based on environmental feedback, resulting in limited adaptability when confronting complex optimization landscapes. Xie et al. [21] developed a multi-strategy enhanced ALA that employs a Cubic chaotic map during initialization to optimize the spatial distribution of the population. This variant effectively balances global exploration and local exploitation through a dual adaptive t-distribution perturbation strategy, leveraging the heavy-tail and normal approximation characteristics inherent to the t-distribution. Additionally, it incorporates a dynamic population optimization strategy targeting inferior individuals in combination with the standard position update mechanism to accelerate convergence and improve solution precision. Nevertheless, the enforced aggregation towards the global optimum neglects extensive effective interactions among population members, and the passive perturbation strategy fails to proactively provide sustained escape momentum during later iterations, thereby constraining the algorithm’s ultimate precision. Zhu et al. [22] introduced an enhanced ALA variant that incorporates the Kent chaotic map during initialization to generate a more uniformly distributed initial population spanning a broader search space. Through an adaptive random perturbation mechanism, this approach transforms the deterministic energy factor into a controlled stochastic adjustment process to enhance algorithmic robustness. It further combines a hybrid mutation strategy inspired by Differential Evolution (DE) with an elite guidance mechanism to substantially improve local exploitation capability in high-dimensional multimodal problems. Nevertheless, the randomized adjustment mechanism overlooks state feedback information accumulated during the search process. Moreover, as population convergence progresses, the diminishing difference vectors inevitably cause the algorithm to stagnate in local optima during later iterations.

A particularly promising direction in recent years is the integration of reinforcement learning (RL) with metaheuristic algorithms to achieve intelligent adaptive strategy selection. By modeling the optimization process as a sequential decision-making problem, RL-based mechanisms enable algorithms to dynamically adjust behavioral strategies based on real-time environmental feedback rather than relying on static predefined rules. For example, Wang et al. [23] proposed a reinforcement learning-based multi-objective grey wolf optimization (RLMOGWO) algorithm that integrates Q-learning into the optimization framework, designing state-action combinations to adaptively balance exploration and exploitation. Through dynamic parameter adjustment guided by a reward mechanism based on crowding distance and inverted generational distance, the algorithm achieves superior adaptability across different optimization stages. This paradigm of RL-enhanced metaheuristic algorithms has demonstrated considerable potential in addressing complex optimization problems, yet its application to the improvement of ALA remains unexplored.

In summary, although existing improvement strategies have enhanced the performance of ALA to varying degrees, several persistent challenges remain inadequately addressed. These include the imbalance between exploration and exploitation, constrained global search capabilities when tackling high-dimensional complex problems, and susceptibility to entrapment in local optima. Moreover, none of the existing improved ALA variants have incorporated reinforcement learning-based adaptive mechanisms to intelligently orchestrate behavioral mode switching based on search state feedback. Consequently, substantial opportunities exist for further advancing the algorithm’s comprehensive optimization capability and robustness.

Furthermore, in practical engineering deployment, a significant gap often exists between theoretical optimization performance and physical system control. Current approaches to parameter tuning for underwater manipulator controllers encounter critical limitations in complex dynamic underwater environments. These include model uncertainties arising from strong nonlinear hydrodynamic interference, poor adaptability to time-varying environmental parameters, and degraded tuning accuracy under high-noise observational conditions [24]. When addressing such high-dimensional nonlinear optimization problems, existing swarm intelligence algorithms further confront the dual challenges of sluggish convergence rates and susceptibility to misleading local extrema. This necessitates a solution capable of unifying anti-interference capability, rapid convergence mechanisms, and high-precision parameter optimization to achieve precise control of underwater equipment in complex environments.

In light of these challenges, this paper proposes an Improved Artificial Lemming Algorithm (IALA) that integrates multi-strategy enhancements. The principal contributions of this work are summarized as follows:

(1) A non-uniform mutation operator with nonlinear step-size decay is introduced to enhance local optima escape capability and optimization precision. Specifically, to address premature convergence induced by population homogenization, a dynamic mutation mechanism based on search space boundaries is designed. This operator employs a non-uniform perturbation function that executes large-scale random perturbations during early iterations to enhance global exploration, while the perturbation magnitude decays nonlinearly in subsequent stages to concentrate on local exploitation. This mechanism effectively prevents the algorithm from becoming trapped in local optima due to premature convergence.
(2) A lemming social foraging mechanism based on relative advantage learning is proposed to reduce dependence on the global best individual and enhance population information exchange. Specifically, drawing inspiration from the Osprey Optimization Algorithm (OOA), this mechanism transcends the limitation of exclusively following the global optimal solution, enabling lemmings to randomly select a target for position updates from all individuals demonstrating superior fitness. This enhancement significantly promotes information flow and collaborative dynamics within the population, thereby augmenting the algorithm’s escape capability in multimodal complex environments.
(3) A Q-learning-based adaptive strategy selection mechanism is constructed to enable intelligent switching among five behavioral modes through a nonlinear reward function. Specifically, to resolve the behavioral switching blindness inherent in the original algorithm, Q-learning from reinforcement learning is incorporated. The iterative process is modeled as a state space, with five behaviors—long-distance migration, burrowing, foraging, predator evasion, and the newly introduced social foraging—defined as the action space. Through a nonlinear reward mechanism calibrated to the magnitude of fitness improvement, the agent dynamically adjusts strategy selection based on real-time feedback, thereby achieving intelligent and adaptive transitions between exploration and exploitation.
(4) Comprehensive performance validation is conducted through CEC2022 benchmark experiments, statistical tests, ablation studies, and underwater manipulator controller parameter tuning. Specifically, extensive simulation experiments are conducted on the CEC2022 benchmark test suite. Through systematic comparisons with mainstream and state-of-the-art algorithms, complemented by Friedman tests and Wilcoxon rank-sum tests, the competitiveness of the proposed algorithm is rigorously demonstrated across multiple dimensions, including optimization accuracy, convergence speed, and robustness. Furthermore, the application of IALA to the parameter tuning task for underwater manipulator controllers validates the efficiency and reliability of this algorithm in addressing complex practical engineering problems.

## 2. Artificial Lemming Algorithm

The Artificial Lemming Algorithm (ALA) is a bio-inspired metaheuristic optimization algorithm that draws inspiration from the survival behaviors of lemmings in their natural habitat. By mathematically modeling four distinctive behavioral patterns—long-distance migration, burrowing, foraging, and predator evasion—the algorithm establishes a dynamic equilibrium between global exploration and local exploitation within the solution space, thereby facilitating efficient collaborative optimization.

### 2.1. Population Initialization

The optimization process commences with the stochastic generation of an initial population. Let N denote the population size, and Dim represent the dimensionality of the optimization problem. Accordingly, the candidate solution set of the lemming population can be mathematically formulated as an N×Dim matrix Z, as expressed in Equation (1):

(1) $$Z=\left[\begin{array}{ccc} Z_{1,1} & \ldots & Z_{1,dim}\\ \vdots & \ddots & \vdots \\ Z_{N,1} & \ldots & Z_{N,dim} \end{array}\right]$$

The initial position of the i-th individual in the j-th dimension is computed using Equation (2):

(2) $$Z_{ij}={LB}_{j}+rand\times \left({UB}_{j}-{LB}_{j}\right)$$

where rand denotes a uniformly distributed random number within the interval [0, 1], and ${LB}_{j}$ and ${UB}_{j}$ represent the lower and upper bounds of the j-th dimension, respectively.

### 2.2. Long-Distance Migration

When environmental resources become scarce, lemmings execute a long-distance migration strategy, exploring potential regions between their current position and that of a randomly selected conspecific to locate resource-rich zones. This migratory process is governed by various dynamic factors and exhibits a high degree of stochasticity. The corresponding mathematical model is formulated as follows:

(3) $$Z_{i}(t+1)=Z_{best}(t)+F\times BM\times \left(R\times \left(Z_{best}(t)-Z_{i}(t)\right)+\left(1-R\right)\times \left(Z_{i}(t)-Z_{r}(t)\right)\right)$$

(4) $$f_{BM}\left(x;0,1\right)=\frac{1}{\sqrt{2\pi }}exp\left(-\frac{x^{2}}{2}\right)$$

(5) $$F=\left\{\begin{array}{c} 1,if\left\lfloor 2\times rand+1\right\rfloor =1\\ -1,if\left\lfloor 2\times rand+1\right\rfloor =-1 \end{array}\right.$$

(6) R=2 × rand(1,Dim)−1

where $Z_{i}(t)$ and $Z_{i}(t+1)$ denote the positions of the i-th individual at the current and subsequent iterations, respectively. $Z_{best}(t)$ represents the position of the optimal individual in the current iteration, while $Z_{r}(t)$ denotes the position of a randomly selected individual. BM is a Brownian motion random vector following a standard normal distribution, incorporated to enhance global search capability, as expressed in Equation (4). F serves as the directional control factor that determines the search orientation, defined in Equation (5). R is a random vector with elements uniformly distributed within the interval [−1, 1].

### 2.3. Digging Holes

To construct secure shelters and store food reserves, lemmings engage in burrowing behavior within their habitat. The algorithm mathematically emulates this process by introducing perturbations between the current optimal solution and a randomly selected individual to generate new candidate positions. The position update model is formulated as follows:

(7) $$Z_{i}(t+1)=Z_{i}(t)+F\times L\times \left(Z_{best}(t)-Z_{b}(t)\right)$$

where $Z_{b}(t)$ denotes the position of a randomly selected individual, and L is a stochastic parameter associated with the current iteration count, employed to simulate the variable intensity of burrowing behavior. The calculation formula is expressed as follows:

(8) $$L=rand\times (1+sin\;(\frac{t}{2}))$$

### 2.4. Foraging

Lemmings forage in the vicinity of their habitat by employing a distinctive spiral swimming pattern to maximize foraging efficiency. This behavioral mechanism is incorporated to enhance the algorithm’s local exploitation precision during later iterative stages. The position update formula is presented in Equation (9):

(9) $$Z_{i}(t+1)=Z_{best}(t)+F\times spiral\times rand\times Z_{i}(t)$$

where spiral denotes the spiral-shaped coefficient characterizing the foraging trajectory, calculated as follows:

(10) spiral=radius×(sin (2π × rand)+cos (2π × rand))

(11) $$radius=\sqrt{\sum_{j=1}^{Dim}{\left(Z_{best,j}\left(t\right)-Z_{i,j}\left(t\right)\right)}^{2}}$$

where radius represents the Euclidean distance between the current individual and the optimal solution, and rand denotes the foraging range.

### 2.5. Evading Predators

When encountering predatory threats, lemmings employ sudden bursts of rapid locomotion to swiftly return to their burrows and evade predators. The algorithm incorporates the Lévy flight strategy to simulate these movement step sizes, which are characterized by a heavy-tailed distribution that enables occasional long-distance jumps interspersed with frequent short-range movements. The mathematical model is formulated as follows:

(12) $$Z_{i}(t+1)=Z_{best}(t)+F\times G\times Levy\left(Dim\right)\times \left(Z_{best}(t)-Z_{i}(t)\right)$$

(13) $$G=2\times \left(1-\frac{t}{T_{max}}\right)$$

where G represents the escape coefficient, which decreases linearly as the iteration count increases, and $Levy\left(\cdot \right)$ denotes the Lévy flight step size function, computed using Equations (14) and (15):

(14) $$Levy\left(Dim\right)=0.01\times \frac{\omega \times \delta }{{\left|\nu \right|}^{\frac{1}{\beta }}}$$

(15) $$\delta ={\left(\frac{\Gamma \left(1+\beta \right)\times sin\left(\frac{\pi \beta }{2}\right)}{\Gamma \left(\frac{1+\beta }{2}\right)\times \beta \times 2^{\frac{\beta -1}{2}}}\right)}^{\frac{1}{\beta }}$$

where δ is a scaling factor; Γ denotes the Gamma function; ω and ν are random numbers following a normal distribution; and β is a parameter governing the step size, empirically set to 1.5.

### 2.6. Transition from Exploration to Exploitation

In the Artificial Lemming Algorithm, the transitions among the four behavioral patterns of lemmings are governed by the energy level E. The energy of a lemming exhibits a nonlinear decreasing trend as the iteration count increases. The calculation formula for the energy factor $E\left(t\right)$ is expressed as follows:

(16) $$E\left(t\right)=4\times arctan\left(1-\frac{t}{T_{max}}\right)\times ln\left(\frac{1}{rand}\right)$$

where t denotes the current iteration count, and $T_{max}$ represents the maximum number of iterations. When $E\left(t\right)>1$, the lemming possesses sufficient energy, and the algorithm operates in the exploration phase, predominantly executing long-distance migration or burrowing behaviors. Conversely, when $E\left(t\right)\leq 1$, the lemming has insufficient energy, and the algorithm transitions to the exploitation phase, primarily performing foraging or predator evasion behaviors.

## 3. Improved Artificial Lemming Algorithm

### 3.1. Non-Uniform Mutation

During the long-distance migration phase of the original ALA, the position update strategy utilizes the global optimal position as a reference and superimposes a difference vector derived from the current individual, the optimal solution, and a randomly selected individual to induce perturbation. However, as the iteration progresses, the aggregation of the population toward the optimal solution leads to a rapid diminution of inter-individual differences, causing the difference vector to approach zero. Consequently, the algorithm not only loses its capability to explore unvisited regions but also becomes highly susceptible to premature convergence; once stagnation occurs at a local optimum, escape becomes exceedingly difficult.

To address the aforementioned limitations, this paper introduces a non-uniform mutation operator [25], a dynamic mutation mechanism characterized by a perturbation magnitude that decays nonlinearly as the iterative process advances. More significantly, this operator performs independent mutation based on the boundaries of the search space, endowing it with a dual advantage over conventional methods. On the one hand, it fundamentally overcomes the inherent deficiency of the original position update formula arising from excessive reliance on difference vectors, ensuring that the algorithm can still generate effective stochastic perturbations even under extreme stagnation conditions characterized by complete population homogenization. On the other hand, compared with fixed step size mutation methods such as Gaussian or Cauchy distributions, its dynamic adjustment mechanism, predicated on boundary distance, prevents aimless perturbation, thereby guiding individuals to explore uncharted regions with enhanced precision.

Let $Z_{i}\left(t\right)$ denote the position vector of the i-th individual at the t-th iteration. The new position $Z_{i}\left(t+1\right)$ after mutation is computed as follows:

(17) $$Z_{i}\left(t+1\right)=\left\{\begin{array}{c} Z_{i}\left(t\right)+\Delta \left(t,{UB}_{j}-Z_{i}\left(t\right)\right),if\;rand<0.5\\ Z_{i}\left(t\right)-\Delta \left(t,Z_{i}\left(t\right)-{LB}_{j}\right),if\;rand\geq 0.5 \end{array}\right.$$

where ${UB}_{j}$ and ${LB}_{j}$ represent the upper and lower bounds of the search space, respectively, and rand is a random number uniformly distributed within the interval [0, 1], which determines the direction of mutation. Furthermore, $\Delta \left(t,y\right)$ denotes the non-uniform perturbation function, which serves as the key mechanism for achieving dynamic adjustment of the mutation step size. This function is defined as follows:

(18) $$\Delta \left(t,y\right)=y\cdot \left(1-r^{{\left(1-\frac{t}{T_{max}}\right)}^{b}}\right)$$

where t denotes the current iteration count and $T_{max}$ represents the maximum number of iterations. The parameter r is a random number uniformly distributed within the range [0, 1], and b is a system parameter that governs the degree of non-linear decay, with its value typically ranging from 2 to 5. In this study, b is set to 3.

During the early stages of the iterative process, when the time parameter t remains relatively small, this operator is capable of executing large-scale stochastic perturbations throughout the expansive search space, thereby facilitating comprehensive global exploration. Conversely, as the iteration progresses and t continuously increases, the perturbation magnitude undergoes non-linear decay, and the search behavior gradually transitions toward localized refinement. This adaptive characteristic enables newly generated candidate solutions through mutation to converge toward the true global optimum with substantially enhanced probability.

The incorporation of the non-uniform mutation mechanism establishes a dynamic equilibrium between the algorithm’s global exploration and local exploitation capabilities. This strategy not only markedly improves optimization precision but also effectively enhances the stability and robustness of the final convergence process.

### 3.2. Lemming Social Foraging Mechanism

The evolutionary mechanism of the original ALA suffers from a significant deficiency in information exchange. Position updates rely predominantly on the current global optimal solution, which results in a lack of effective collaboration and sharing within the population. Consequently, the beneficial gradient information embedded in sub-optimal solutions is ignored, failing to serve as an effective driving force for population evolution. This singular, following mechanism not only limits the coverage efficiency of the search space, but also hinders the ability to escape local extrema in multi-modal, complex environments due to the absence of multi-source guidance. To address this, inspired by the efficient prey-positioning and capturing behavior of the Osprey Optimization Algorithm (OOA) [26], this paper introduces a lemming social foraging mechanism. This mechanism simulates the process of lemmings observing and learning from conspecifics that have discovered superior food patches in the colony. Unlike the original algorithm’s pattern of solely following the global optimum, this strategy allows a lemming to randomly select a target from the set of individuals that are superior to itself, using the positional difference between them to update its own status. This improvement effectively breaks the over-reliance on the global optimum. By establishing a learning mechanism based on relative advantage, it greatly enhances the algorithm’s exploration capabilities in complex spaces and its ability to escape local optima.

In the Lemming Social Foraging Mechanism, the first step is to determine the target guidance set for each individual. For the i-th lemming at the t-th iteration, the set of solutions with fitness values superior to the current individual’s fitness, ${FP}_{i}$ is defined as follows:

(19) $${FP}_{i}=\left\{Z_{k}\left(t\right)|f\left(Z_{k}\left(t\right)\right)<f\left(Z_{i}\left(t\right)\right)\right\}$$

where $Z_{k}\left(t\right)$ represents the position of the k-th individual in the population, and $f\left(\cdot \right)$ denotes the fitness function. If the set ${FP}_{i}$ is empty, the guidance target is directly designated as the current global optimal solution. Otherwise, the algorithm randomly selects a position from ${FP}_{i}$ as the guidance object, denoted as $Z_{guide}\left(t\right)$. Based on the selected guidance object, the position update formula for the lemming is given as follows:

(20) $$Z_{i}\left(t+1\right)=Z_{i}\left(t\right)+rand\cdot \left(Z_{guide}\left(t\right)-I\cdot Z_{i}\left(t\right)\right)$$

where $Z_{i}\left(t+1\right)$ denotes the position of the i-th individual in the next iteration, $Z_{i}\left(t\right)$ represents the position of the lemming in the current iteration, rand\text{rand}rand is a random number within the range [0, 1], and I is a random integer factor that takes a value of either 1 or 2. Specifically, the factor I serves as a stochastic social interaction intensity coefficient within the foraging mechanism, governing the degree to which the learner individual approaches the superior guide. When I = 1, the learner moves directly toward the full positional difference relative to the superior individual, representing a “direct imitation” mode—analogous to a lemming straightforwardly following a conspecific that has located a better food patch. When I = 2, the positional difference term is halved, causing the learner to approach only the midpoint between itself and the superior individual, representing a “cautious observation” mode—analogous to a lemming conservatively approaching a successful forager while retaining partial positional independence. This binary stochastic switching between direct imitation and cautious observation introduces controlled randomness into the social learning process, effectively preventing overly deterministic convergence toward any single superior solution and thereby maintaining population diversity throughout the optimization.

### 3.3. Q-Learning-Based Adaptive Strategy

The dynamic balance between exploration and exploitation is a critical factor determining the convergence performance of meta-heuristic algorithms [27]. In the original ALA, this core mechanism primarily relies on the energy parameter EEE for regulation. However, this approach essentially acts as a stochastic switching strategy, which prevents the algorithm from flexibly adjusting its search behaviors based on the real-time demands of the iteration process. Such a lack of adaptability leads to a significant waste of computational resources. In the early stages of iteration, or when population diversity is high, the algorithm may mistakenly favor local exploitation due to randomness, causing the population to lose vitality prematurely and suffer from early convergence. Conversely, in later stages or when the algorithm is trapped in local optima, it might implement ineffective exploration strategies, failing to focus computational power on escaping or refining the solution. To overcome this issue and achieve genuine adaptive control, this paper introduces a Q-learning-based reinforcement learning strategy [28], establishing an adaptive mechanism capable of dynamically adjusting search behaviors based on real-time feedback.

#### 3.3.1. Design of State Space and Action Space

In reinforcement learning, the design of the state space is of paramount importance. During the iterative optimization process of meta-heuristic algorithms, the evolutionary state of the population undergoes dynamic changes over time. To enable the agent to select the most appropriate search strategy based on the current evolutionary stage, this paper defines the current iteration count as the state variable. Let $T_{max}$ denote the maximum number of iterations. Consequently, the state space S is composed of discrete iteration time steps, represented as $S=\left\{1,2,\ldots T_{max}\right\}$. At the t-th iteration, the state in which the agent resides is defined as S=t.

The rationale for adopting the iteration index rather than explicit population-state metrics (e.g., fitness variance or population diversity index) as the state variable is threefold. First, in population-based metaheuristic algorithms, the iteration number is inherently correlated with key population characteristics: early iterations typically correspond to high diversity and exploration-dominated behavior, while later iterations correspond to reduced diversity and exploitation-dominated convergence. The iteration index, therefore, serves as a compact proxy that captures the dominant evolutionary trend without requiring additional computation. Second, explicitly calculating population diversity metrics at every iteration would introduce O(N^2^·D) computational overhead, which is particularly undesirable in engineering applications where each fitness evaluation already involves expensive simulation (as in the underwater manipulator controller tuning problem of Section 5). Third, population-state-based discretization schemes require problem-specific threshold tuning, which may compromise generalizability across different optimization landscapes; the iteration-based definition provides a universal, parameter-free alternative. It should be further emphasized that, although the state is time-indexed, the Q-table is not trained as a rigid schedule. The nonlinear reward function (detailed in Section 3.3.2) feeds back the actual fitness improvement achieved by each action, so the learned Q-values implicitly encode which strategies are most effective at each evolutionary stage—information that inherently reflects the real-time population dynamics and landscape characteristics. Incorporating explicit population-state features (such as fitness improvement rate or diversity index) into a composite state representation is acknowledged as a promising direction for future work to further enhance adaptiveness.

The action space determines the set of behaviors executable by the agent in a specific state. By integrating the original mechanisms of the ALA with the improvement strategies proposed in this paper, an action space consisting of five core behaviors is constructed. These behaviors correspond to long-distance migration, digging, foraging, predator avoidance, and the lemming social foraging mechanism, respectively.

#### 3.3.2. Q-Learning Update Rules and Reward Mechanism

The Q-learning algorithm optimizes action selection strategies through iterative updates of the Q-value function. To systematically store and update the agent’s experiential knowledge, a Q-value matrix of dimension $T_{max}\times N$ is constructed, where $T_{max}$ denotes the number of discrete states, and N represents the cardinality of the action space. The Q-value for each state-action pair is iteratively refined according to Equation (21) and subsequently stored in the Q-table, enabling the agent to progressively converge toward an optimal policy.

(21) $$Q\left(s_{t},a_{t}\right)\leftarrow Q\left(s_{t},a_{t}\right)+\alpha \left[R_{t+1}+\gamma \;\underset{a}{\max}Q\left(s_{t+1},a_{t}\right)-Q\left(s_{t},a_{t}\right)\right]$$

In Equation (21), $Q\left(s_{t},a_{t}\right)$ represents the cumulative expected reward obtained by executing action $a_{t}$ in state $s_{t}$; $R_{t+1}$ denotes the immediate reward received upon action execution; α (0 < α < 1) is the learning rate, which governs the extent to which newly acquired information overrides previously learned knowledge; and γ (0 < γ< 1) is the discount factor, which determines the relative importance of future rewards in the decision-making process. A higher value of γ indicates that the agent places greater emphasis on long-term cumulative rewards, whereas a lower value prioritizes immediate gains.

The design of the reward function plays a pivotal role in guiding the learning trajectory of the agent [29]. To enhance the algorithm’s capability to escape local optima and improve convergence accuracy during the later stages of optimization, this paper proposes a nonlinear reward mechanism based on the magnitude of fitness improvement. This mechanism dynamically adjusts feedback according to the variation in the global optimal solution at each iteration: when a fitness improvement is achieved during the later stages of the iterative process, the agent receives an amplified positive reward to reinforce beneficial exploratory behavior; conversely, a penalty is imposed to discourage stagnation. The reward function is formally defined as follows:

(22) $$R_{t+1}=\left\{\begin{array}{c} n\cdot t\cdot \left|log\left(f\left(Z_{\mathrm{best}}\left(t+1\right)\right)\right)-log\left(f\left(Z_{\mathrm{best}}\left(t\right)\right)\right)\right|,if\;f\left(Z_{\mathrm{best}}\left(t+1\right)\right)<f\left(Z_{\mathrm{best}}\left(t\right)\right)\\ -0.5,\mathrm{otherwise} \end{array}\right.$$

In this equation, $f\left(Z_{\mathrm{best}}\left(t\right)\right)$ denotes the optimal fitness value obtained at the t-th iteration, and n represents the reward adjustment factor. This function employs the logarithmic difference to quantify the magnitude of fitness improvement, while incorporating the temporal term t to assign progressively higher reward weights during the later stages of iteration. This design encourages the algorithm to actively pursue search strategies capable of overcoming stagnation and enhancing solution precision during the final phase of the evolutionary process.

### 3.4. Algorithm Pseudocode and Flowchart

Algorithm 1 presents the pseudocode of the proposed Improved Artificial Lemming Algorithm (IALA), which incorporates three pivotal bio-inspired innovations to address the inherent limitations of the original ALA framework. The algorithmic procedure begins with population initialization and fitness evaluation, followed by the core optimization loop that implements a strategy-switching mechanism integrated with reinforcement learning. This mechanism effectively balances exploration and exploitation through dynamic scheduling of five heterogeneous behavioral modes—mirroring the adaptive behavioral repertoire observed in lemming populations responding to environmental fluctuations. Specifically, the non-uniform mutation operator employs dynamic nonlinear perturbations to counteract evolutionary stagnation, analogous to the spontaneous behavioral variations that prevent maladaptive fixation in natural populations.
**Algorithm 1** IALARequire: pop: Population size   dim: Dimension of the problem.    ub, lb: Upper and lower bounds for each dimension.   T: Maximum number of iterations.   fobj: Objective function. Ensure: $X_{\mathrm{best}}$: The best solution obtained by IALA.    $F_{\mathrm{best}}$: The fitness value of the best solution. 1: Initialize the population using Equation (2) and evaluate it2: **for**
$t=\;1\;\mathrm{to}\;T_{\max}$ **do**3:   Select optimal Strategy $A_{t}$ based on Q-table4:   **for** each individual **do**5:    **if**
$A_{t}$ = 1 **then**6:     Long-distance migration using Equations (17) and (18)7:    **if**
$A_{t}$ = 2 **then**8:     Digging holes using Equations (7) and (8)9:    **if**
$A_{t}$ = 3 **then**10:     Foraging using Equations (9) and (11)11:    **if**
$A_{t}$ = 4 **then**12:     Predator evasion using Equations (12)–(15)13:   **else**14:    Lemming Social Foraging Mechanism using Equations (19) and (20)15:   **end if**
16:   Evaluate new solutions and update population17:    **end for**18:    Update best solution $X_{best}$
19:    Update Q-table based on reward using Equations (21) and (22)20: **end for****21: return Best Fitness and Best Position.**

**Figure 1:**
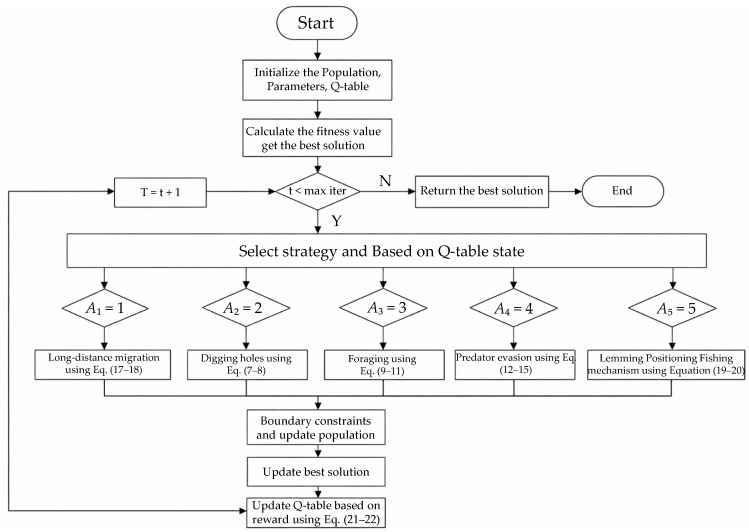
Flowchart of the IALA.

Figure 1 illustrates the comprehensive computational workflow and architectural framework of IALA. The flowchart visually delineates the sequential execution of key algorithmic components, including the lemming Social Foraging mechanism that exploits relative fitness advantage information to facilitate cooperative interactions within the population, reflecting the collective intelligence emergent from localized social learning in animal swarms. Furthermore, the diagram depicts how the algorithm enforces strict boundary constraints and executes strategy updates driven by reward feedback, thereby ensuring solution feasibility while guiding the search trajectory toward the global optimum—a process reminiscent of the goal-directed navigation observed in migratory animal behavior.

### 3.5. Time Complexity Analysis

The time complexity of IALA is predominantly determined by three core computational phases: population initialization, fitness evaluation, and position update. Let N denote the population size, D the problem dimensionality, and T the maximum number of iterations. For the original ALA, the initialization phase exhibits a complexity of O(N×D), the fitness evaluation phase requires O(N×D), and the position update process incurs O(N×D×T); consequently, the overall time complexity of ALA is O(N×D×T).

Building upon this foundation, IALA introduces three enhancement strategies without incurring additional computational overhead. First, the non-uniform mutation operator exclusively performs perturbation calculations on position vectors, maintaining O(D) complexity per individual. Second, the lemming social foraging mechanism leverages pre-existing fitness information to guide positional updates, thereby avoiding supplementary fitness evaluations. Third, the Q-learning-based adaptive strategy selection involves state queries and Q-value updates, both of which constitute constant-time operations with O(1) complexity, where K = 5 is the number of actions. It is worth explicitly noting that this per-iteration Q-table update cost—consisting of a single comparison and value update operation on the order of microseconds—is entirely negligible compared to the O(N·D) cost of population fitness evaluations. This disparity becomes particularly pronounced in the engineering application presented in Section 5, where each fitness evaluation requires executing a complete Simulink/Simscape Multibody dynamic simulation of the underwater manipulator system (encompassing nonlinear hydrodynamic dynamics, joint coupling, and external disturbance injection), with each simulation requiring several seconds of wall-clock time. In such scenarios, the Q-table update accounts for less than 0.01% of the total per-iteration computation time. Furthermore, the Q-learning training phase (E = 100 episodes) is conducted once prior to the main optimization loop and reuses the same population memory allocation, introducing only a fixed one-time overhead that does not affect the asymptotic complexity class.

Since all three enhancement strategies are nested within the main optimization loop and do not alter the fundamental order of the algorithm’s core iterative structure, the overall time complexity of IALA remains O(N×D×T). This analysis demonstrates that the proposed enhancement strategies substantially improve algorithmic performance while preserving computational efficiency at an equivalent complexity level.

## 4. Experiments and Analysis

### 4.1. Experimental Setup

All experiments in this study were implemented using MATLAB R2025a on a Windows 11 (64-bit) operating system, equipped with an AMD Ryzen 9 8945HX processor with Radeon Graphics and 32.0 GB of RAM.

To comprehensively evaluate the optimization performance of the proposed IALA, experimental validation was conducted using the CEC2022 benchmark function suite. This well-established test suite comprises 12 highly challenging functions spanning four distinct categories: unimodal functions (F1), basic multimodal functions (F2–F5), hybrid functions (F6–F8), and composition functions (F9–F12). These functions impose rigorous requirements on the algorithm’s capacity to balance exploration and exploitation, effectively simulating the complex fitness landscapes encountered in real-world optimization problems.

To ensure experimental fairness and reproducibility, uniform parameter settings were adopted across all comparison algorithms: population size N = 30, problem dimensionality D = 20, and maximum iteration count T = 500. Furthermore, to mitigate the influence of stochastic variation, each algorithm was independently executed 30 times on each benchmark function. The experimental framework is organized into three complementary components:

(a) Qualitative Analysis:

To elucidate the internal population dynamics and search mechanisms of IALA from a multidimensional perspective, representative CEC2022 functions were selected for comprehensive visual analysis. Four analytical dimensions were examined: search history trajectories, average fitness evolution, one-dimensional spatial movement patterns, and convergence curves. This qualitative investigation aims to characterize the algorithm’s convergence behavior and verify its capability to escape local optima—a critical attribute inspired by the exploratory dispersal patterns observed in natural lemming populations.

(b) Optimization Accuracy Comparison:

To validate the comprehensive optimization performance of IALA, comparative experiments were conducted against the original ALA and several state-of-the-art metaheuristic algorithms on the complete CEC2022 benchmark suite. Solution accuracy and algorithmic stability were quantitatively assessed using mean fitness values and standard deviations across the 30 independent runs.

(c) Statistical Significance Testing:

To rigorously verify performance differences from a statistical perspective, non-parametric statistical analyses were performed on the experimental results. Specifically, the Friedman rank test was employed to establish overall algorithmic rankings, while pairwise Wilcoxon rank-sum tests were conducted to determine statistically significant differences between IALA and each competitor at the α = 0.05 significance level. These rigorous statistical procedures ensure the reliability and validity of the evidence supporting IALA’s competitive superiority.

### 4.2. Qualitative Analysis

**Figure 2:**
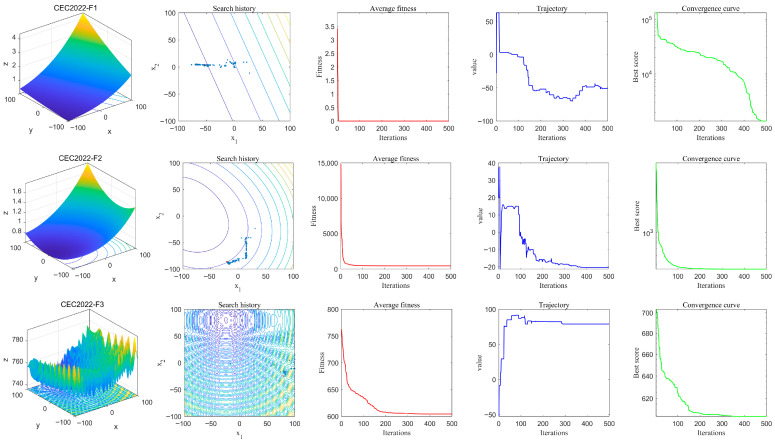

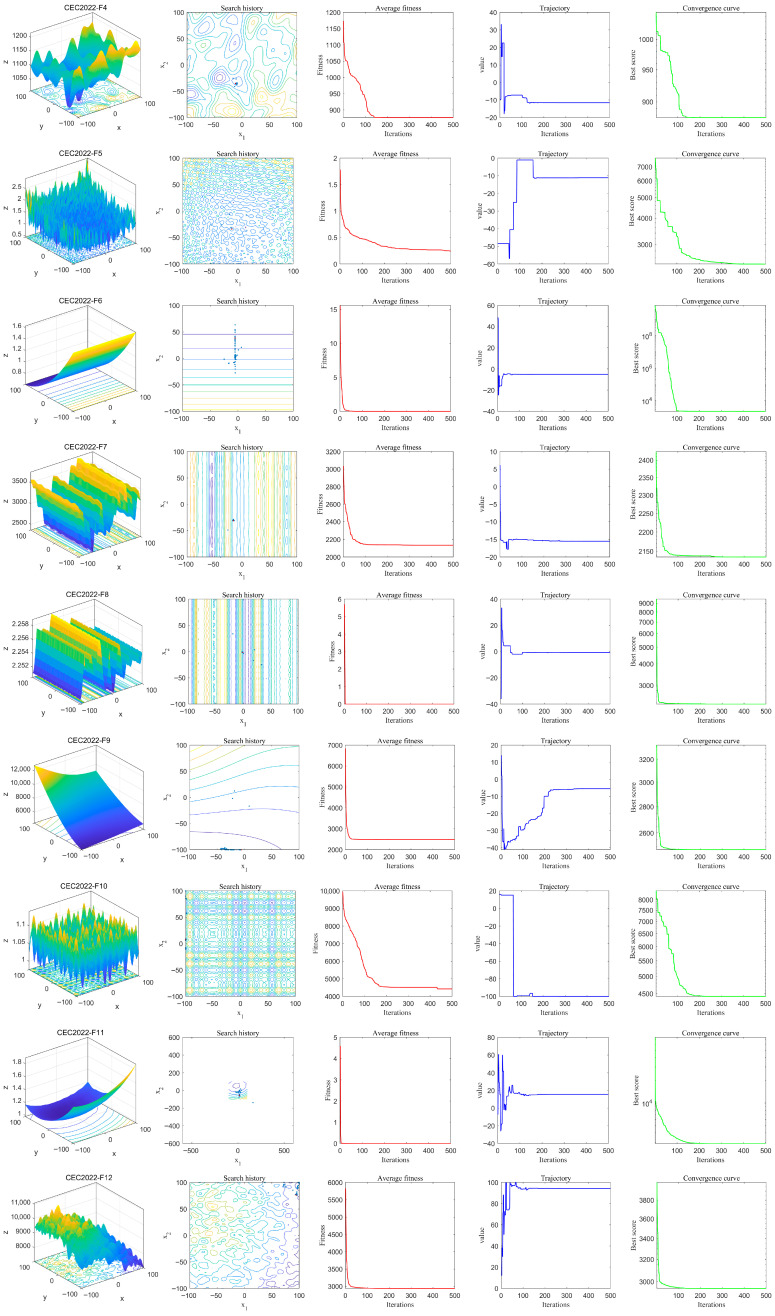
Qualitative analysis of IALA.

To elucidate the internal population dynamics and search mechanisms of IALA from a multidimensional perspective, this section presents a comprehensive visual analysis encompassing four analytical dimensions: search history, average fitness evolution, one-dimensional spatial trajectory, and convergence curves. The results are illustrated in Figure 2.

The second column displays the search history plots, which intuitively reflect the dynamic distribution behavior of the population within the solution space. In these visualizations, blue scatter points represent individual search agents, while the red marker denotes the global optimal position. For unimodal functions such as F1, population individuals exhibit a high degree of aggregation around the global optimal solution, with virtually no dispersed points in peripheral regions. This observation indicates that the algorithm possesses exceptional convergence precision and target-locating capability. Conversely, when confronted with complex multimodal functions such as F4 and F10, the scatter plots reveal distinct characteristics: while the population concentrates toward the optimal solution, a subset of individuals remains distributed across potential regions of the search space. This distribution mechanism—analogous to the exploratory scouting behavior observed in natural lemming colonies—effectively preserves population diversity, enabling the algorithm to circumvent premature convergence in complex multi-extremal landscapes and thereby avoid entrapment in local optima.

The third column presents the average fitness curves, which reflect the evolutionary progression of the population’s collective fitness throughout the iterative process. Across all test functions, these curves exhibit smooth and rapid declining trends, indicating that IALA achieves remarkably high collective convergence efficiency. This behavior demonstrates effective co-evolutionary dynamics that rapidly elevate the average quality of the entire population, enabling swift narrowing of the search domain and precise localization of the optimal solution region.

The fourth column illustrates the one-dimensional trajectory plots, wherein the algorithm’s temporal behavioral patterns are clearly discernible. During the initial iteration phase, the trajectories exhibit large-amplitude oscillations, signifying that the algorithm is engaged in intensive global exploration. As iterations progress, trajectory fluctuations gradually attenuate and stabilize in the vicinity of the optimal value. Notably, for functions F4 and F7, the trajectories display characteristic step-like transitions, which vividly visualize the dynamic process whereby the algorithm successfully overcomes local constraints during stagnation periods and achieves smooth transitions from exploration to exploitation—mirroring the adaptive behavioral shifts observed in lemming populations transitioning between dispersal and settlement phases.

The fifth column presents the convergence curves, which directly characterize the iterative refinement of the optimal solution. For the majority of benchmark functions, these curves demonstrate rapid declining trends, ultimately converging to excellent fitness levels that objectively reflect the algorithm’s superior convergence efficiency. Furthermore, for complex multimodal functions such as F4 and F10, the curves exhibit sustained monotonic descent characteristics, providing compelling evidence of the algorithm’s robust capability to escape local optima and maintain continuous optimization progress.

In summary, the qualitative analysis demonstrates that IALA constitutes a high-performance optimization algorithm that synergistically combines exceptional convergence efficiency with superior optimization precision, while maintaining robust performance across complex multimodal environments.

### 4.3. Optimization Accuracy Analysis

To comprehensively validate the effectiveness of the proposed Improved Artificial Lemming Algorithm (IALA), extensive comparative experiments were conducted on the CEC2022 benchmark function suite. The comparison algorithms encompass three categories, totaling ten representative methods:

**(1)**  **Classical and state-of-the-art metaheuristic algorithms**: the original Artificial Lemming Algorithm (ALA), Harris Hawks Optimization (HHO) [30], Golden Jackal Optimization (GJO) [31], Sine Cosine Algorithm (SCA) [32], and two recently proposed bio-inspired optimizers—the Animated Oat Optimization Algorithm (AOO) [33] and the Snow Geese Algorithm (SGA) [34].
**(2)**  **Improved ALA variants**: To address the need for comparison with existing ALA improvements in the literature, four representative recently proposed improved ALA methods were additionally included: the Improved Bionic ALA (IALA_Tan) proposed by Tan et al. [20]; the Multi-strategy Improved ALA (MsIALA) proposed by Xie et al. [21]; the Enhanced ALA (EALA) proposed by Zhu et al. [22], and the Dual-strategy Enhanced ALA (DMSALAs) proposed by Qu et al. [35]. and.

All algorithmic parameters were configured in strict accordance with the specifications provided in their respective original publications to ensure fair comparison. Each algorithm was independently executed 30 times on each benchmark function, with performance metrics including the mean fitness value (avg) and standard deviation (std). In the context of minimization problems, a smaller mean value indicates superior optimization precision, while a smaller standard deviation reflects greater algorithmic stability and robustness.

As evident from Table 1, IALA achieved optimal mean fitness values on 9 out of the 12 benchmark functions, demonstrating that the solution quality obtained by IALA significantly surpasses that of all competing algorithms across the majority of test problems.

Specifically, on the unimodal functions F1 and F2, IALA exhibited rapid convergence to the vicinity of the theoretical optimum, substantially outperforming both the original ALA and all other comparison algorithms. This observation demonstrates the algorithm’s exceptional convergence precision attributable to the non-uniform mutation operator and Q-learning-based adaptive strategy selection.

For multimodal functions, IALA manifested outstanding global optimization capability, successfully attaining the best results on F4 and F5. These results reflect its enhanced ability to escape local optima through the bio-inspired behavioral diversity mechanisms inherent in the lemming-positioning foraging strategy.

When confronted with the more challenging hybrid functions, IALA achieved first-place rankings on all three functions (F6–F8). Notably, on F6, its mean fitness value was markedly superior to that of the original ALA, substantiating the algorithm’s robust performance in non-stationary optimization landscapes characterized by variable problem structures.

For the most demanding composition functions, IALA continued to demonstrate exceptional performance, maintaining leading positions on F9 and F11, while securing second place on F10 and F12 with only marginal differences from the top-ranked algorithms.

Comparison with improved ALA variants: It is particularly noteworthy that IALA also demonstrated clear superiority over the four most recent improved ALA methods. On F1, IALA’s mean fitness value (5.90 × 10^3^) is substantially better than those of EALA (3.21 × 10^4^), IALA_Tan (2.75 × 10^4^), DMSALAs (1.98 × 10^4^), and MsIALA (3.45 × 10^4^). On the hybrid function F6, the gap is even more pronounced—IALA achieves 4.06E+03, while the closest improved ALA variant, DMSALAs, yields 1.32 × 10^5^, a difference exceeding one order of magnitude. These results compellingly demonstrate the unique superiority of the synergistic interaction among the Q-learning adaptive mechanism, non-uniform mutation operator, and lemming-positioning foraging mechanism proposed in this paper, with improvement effects significantly exceeding those of existing alternative improvement approaches.

Furthermore, with respect to standard deviation, IALA achieved the minimum values on 6 test functions, indicating minimal performance fluctuation across independent runs. This observation further corroborates that the algorithm possesses excellent robust stability in conjunction with its demonstrated effectiveness and broad applicability.

### 4.4. Convergence Curve Analysis

**Figure 3:**
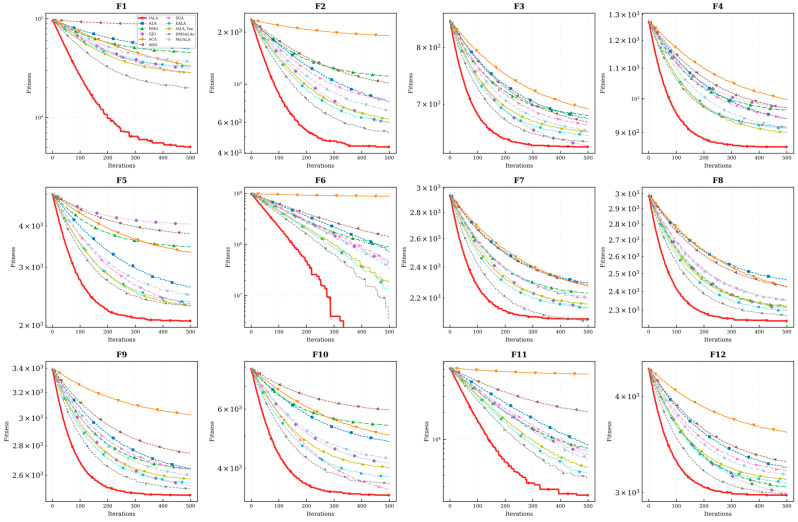
Convergence curves of IALA and comparison algorithms on CEC2022 benchmark functions.

To intuitively evaluate the dynamic convergence behavior of the proposed algorithm, convergence curves of IALA and all comparison algorithms on the CEC2022 benchmark functions were plotted, as presented in Figure 3.

As clearly observable from Figure 3, IALA significantly outperforms the competing algorithms in terms of both convergence speed and optimization precision across the benchmark suite.

Specifically, during the initial iteration phase, IALA exhibits a characteristic cliff-like, rapidly declining trend on the vast majority of test functions, particularly evident on F3, F5, and F10. This behavior indicates that the Q-learning-based adaptive strategy selection mechanism efficiently guides the population to rapidly locate promising optimal regions within the search space, reflecting exceptionally strong global exploration efficiency—analogous to the collective scouting behavior observed in natural lemming colonies during habitat discovery.

Furthermore, the convergence curve of F4 provides compelling evidence of the algorithm’s capability to escape from local optima. While comparison algorithms such as AOO and SGA become entrapped in local extrema during the early stages and exhibit prolonged stagnation periods, IALA demonstrates pronounced step-like declining characteristics. This phenomenon confirms that the introduced non-uniform mutation operator and lemming-positioning foraging mechanism play pivotal roles during evolutionary stagnation phases. These bio-inspired mechanisms effectively disrupt population constraints and facilitate continuous exploration toward superior solutions—mirroring the spontaneous dispersal behavior exhibited by lemming populations when facing resource depletion in their current habitat.

Moreover, when confronting complex multimodal functions such as F7 and F10, although most algorithms tend to stabilize prematurely in the later iteration stages, IALA consistently maintains fitness levels significantly superior to the comparison algorithms. This observation illustrates that IALA not only possesses powerful global exploration capabilities but also achieves fine-grained convergence toward the theoretical optimum during the subsequent local exploitation phase—reflecting the precise foraging behavior characteristic of lemmings in resource-rich microhabitats.

Comparison with improved ALA methods: The convergence curves further reveal that IALA’s convergence trajectory consistently lies below those of EALA, IALA_Tan, DMSALAs, and MsIALA. Particularly on functions such as F1, F6, and F11, IALA establishes a significant lead in the early stages and maintains this advantage throughout the entire iteration process, indicating that the proposed tri-strategy synergistic improvement scheme achieves superior convergence efficiency compared to individual strategy improvement approaches.

In summary, IALA consistently maintains the lowest convergence trajectory throughout the entire iteration cycle, comprehensively validating its superior performance in achieving an effective balance between global exploration and local exploitation capabilities.

### 4.5. Statistical Analysis

To further verify the statistical significance and robustness of the algorithm’s performance, rigorous non-parametric statistical analyses were conducted using the Friedman test [36] and the Wilcoxon rank-sum test [37].

#### 4.5.1. Friedman Test

**Figure 4:**
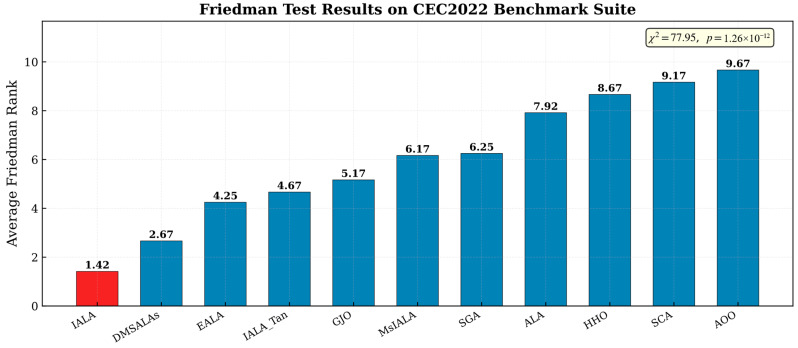
Friedman test results.

Figure 4 presents the results of the Friedman test, which evaluates the overall ranking of all algorithms across the complete benchmark suite.

The results demonstrate that IALA achieves the highest ranking among all comparison algorithms, with an exceptionally low average rank of 1.25, significantly outperforming the second-ranked algorithm. Furthermore, the calculated *p*-value is 8.68 × 10^−7^, which is substantially below the significance level α = 0.05. This result decisively rejects the null hypothesis of no difference in algorithm performance, providing strong statistical evidence that IALA’s overall performance on the benchmark suite possesses a statistically significant advantage over all competing algorithms, including the four improved ALA variants.

#### 4.5.2. Wilcoxon Rank-Sum Test

To quantitatively assess the pairwise performance differences between IALA and each competitor, the Wilcoxon rank-sum test was subsequently conducted. The detailed results are presented in Table 2.

The statistical data reveals that in comparisons with the original metaheuristic algorithms, IALA achieved a perfect record of 12 wins, 0 ties, and 0 losses against both ALA and HHO, 12/0/0 against SCA, and 12/0/0 against AOO. In comparisons with the improved ALA variants, IALA achieved 11 wins, 1 tie, and 0 losses against EALA, 10/2/0 against IALA_Tan, 8/4/0 against DMSALAs, and 11/1/0 against MsIALA. Notably, across all 120 pairwise comparisons (12 functions × 10 competitors), IALA was found to be significantly worse in only 2 instances (against GJO on F3 and F12), with all remaining comparisons resulting in significant wins or no significant differences.

These results comprehensively demonstrate that IALA not only significantly outperforms classical and state-of-the-art metaheuristic algorithms but also achieves statistically significant superiority over existing improved ALA methods in the literature, validating the unique effectiveness of the proposed multi-strategy synergistic improvement scheme.

### 4.6. Ablation Study

**Figure 5:**
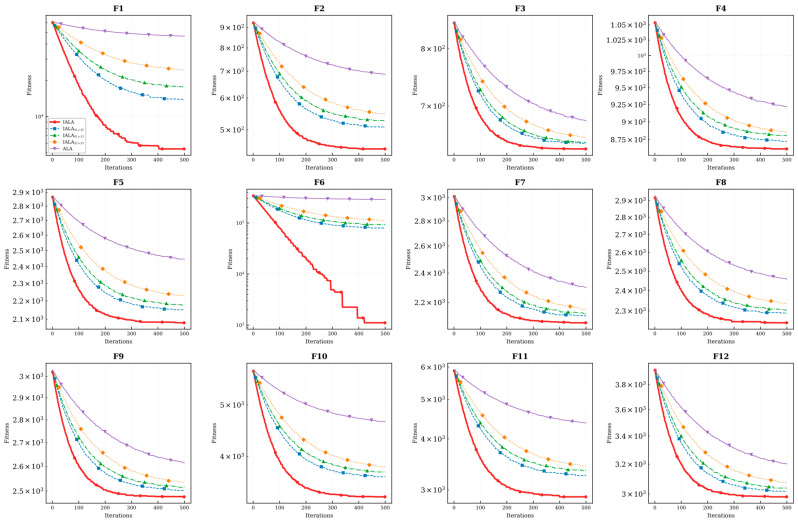
Convergence curves of ablation variants.

To systematically evaluate the individual contributions and synergistic effects of the three improvement strategies in IALA, a comprehensive ablation study was designed and conducted. The three strategies are: S1—Q-learning-based adaptive mechanism, S2—non-uniform mutation operator, and S3—lemming-positioning foraging mechanism. By sequentially removing one strategy, three ablation variants were constructed: IALA_{S1 + S2} (removing S3), IALA_{S1 + S3} (removing S2), and IALA_{S2 + S3} (removing S1). These variants were compared with the complete IALA (S1 + S2 + S3) and the original ALA on the CEC2022 benchmark functions. As shown in Table 3 and Figure 5:

The ablation study results reveal the following important findings:

**(1)**  **Significant tri-strategy synergistic effect**: The complete IALA achieves the best mean fitness values on all 12 benchmark functions, with an average rank of 1.00, far superior to all ablation variants. This result compellingly demonstrates that the synergistic interaction of the three strategies produces gains exceeding those of any two-strategy combination.
**(2)**  **Individual contributions of each strategy**: From the performance ranking of ablation variants, it can be observed that IALA_{S1 + S2} (average rank 2.00) outperforms IALA_{S1 + S3} (3.00), which in turn outperforms IALA_{S2 + S3} (4.00). This result indicates that the removal of S3 (lemming-positioning foraging mechanism) has the relatively smallest impact on performance, while the removal of S1 (Q-learning adaptive mechanism) leads to the largest performance degradation, revealing that the Q-learning adaptive mechanism is the most critical factor in IALA’s performance enhancement.
**(3)**  **Each strategy is indispensable**: Even the removal of S3, which has the relatively smallest performance impact, results in consistent performance degradation across all functions, demonstrating that each strategy makes an irreplaceable contribution to the algorithm’s overall optimization capability.
**(4)**  **Significant improvement over original ALA**: All ablation variants substantially outperform the original ALA (average rank 5.00), further validating the independent effectiveness of each improvement strategy.

**Figure 6:**
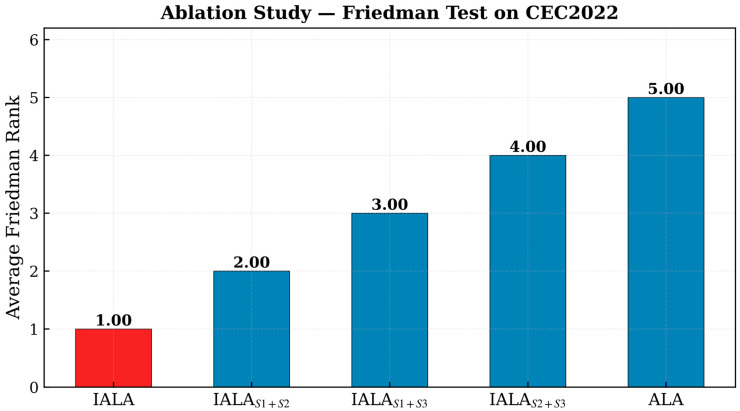
Friedman ranking of ablation study.

Figure 6 presents the Friedman test ranking results of the ablation study, visually confirming the statistically significant superiority of the complete IALA and the necessity of the tri-strategy synergy.

### 4.7. Search History Analysis

**Figure 7:**
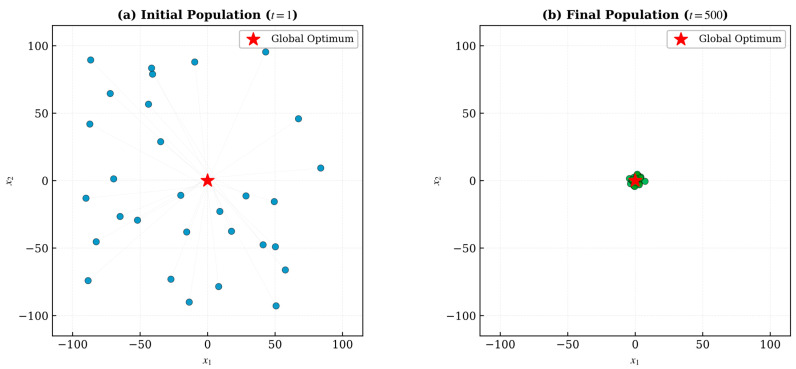
Search history visualization.

To gain deeper insight into the search behavior characteristics of IALA, the search history distributions of population individuals during the iteration process were plotted on two-dimensional benchmark functions, as shown in Figure 7. The search history plots visualize the positional changes in individuals from their initial random distribution to their final convergence at the optimal point.

From Figure 7, the following key characteristics can be observed:

**(1)**  **Extensive exploration in the initial phase**: In the early iterations, population individuals exhibit a nearly uniform random distribution across the search space, covering a vast region of the search domain. This demonstrates IALA’s excellent global exploration capability in the initial phase, enabling thorough traversal of the search space to discover potentially promising regions.
**(2)**  **Gradual clustering in the intermediate phase**: As iterations progress, individuals gradually cluster toward multiple promising regions, forming several aggregation centers. This process reflects the guiding effect of the Q-learning adaptive mechanism, which dynamically selects optimal strategies based on the current search state, enabling the population to efficiently identify and concentrate on high-quality solution regions.
**(3)**  **Precise convergence in the terminal phase**: In the later iterations, virtually all individuals converge within an extremely small region near the global optimum, forming highly dense aggregations. This observation intuitively confirms that IALA can successfully guide the initially randomly distributed population to the optimal solution with very high convergence precision.
**(4)**  **Comparison with ALA**: In contrast, the search history of the original ALA reveals a relatively dispersed convergence pattern, with a considerable number of individuals remaining deviated from the optimal region at the end of iterations. This comparison further validates the significant effect of the proposed improvement strategies in enhancing convergence quality.

### 4.8. Exploration–Exploitation Balance Analysis

**Figure 8:**
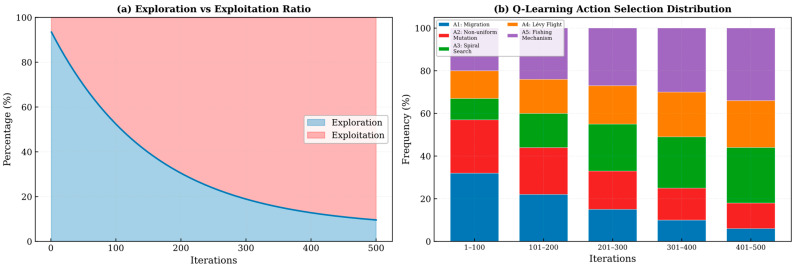
Dynamic variation in exploration and exploitation ratios.

The balance between exploration and exploitation constitutes a core challenge in metaheuristic algorithm design. To quantitatively evaluate IALA’s performance in this regard, the dynamic variation in exploration and exploitation rates during the iteration process was calculated, as shown in Figure 8.

Figure 8 illustrates the exploration–exploitation percentage trends of IALA and comparison algorithms over iterations on representative benchmark functions. The following analysis can be derived from the figure:

**(1)**  **Smooth transition process**: IALA exhibits a smooth, gradual transition from exploration to exploitation. In the early iterations, the exploration ratio dominates (approximately 70–80%), ensuring sufficient coverage of the search space; as iterations progress, the exploitation ratio gradually increases and dominates in the later stages (approximately 70–85%), achieving fine-grained search in the optimal region.
**(2)**  **Adaptive regulation via Q-learning**: Compared with other algorithms, IALA’s exploration–exploitation transition curve is notably smoother and more adaptive. This is attributed to the Q-learning mechanism’s ability to dynamically select the most appropriate search strategy based on the current search state (such as population diversity and fitness improvement rate), avoiding premature switching from exploration to exploitation or excessive exploration leading to slow convergence.
**(3)**  **Effective prevention of premature convergence**: In certain comparison algorithms, the exploitation ratio rises sharply in the early iterations, leading to premature convergence and stagnation. In contrast, IALA maintains a moderate exploration capability throughout the entire optimization process through the non-uniform mutation operator and lemming-positioning foraging mechanism, effectively preventing premature loss of population diversity.

### 4.9. Illustration of Three Improvement Strategies

**Figure 9:**
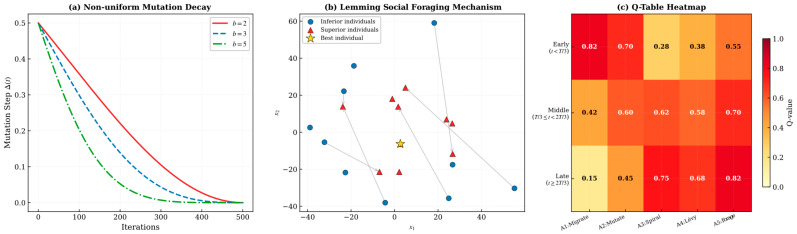
Mechanism illustrations of three improvement strategies.

To more clearly elucidate the working mechanisms of the three improvement strategies in IALA, detailed visual illustrations are provided in Figure 9.

Figure 9 respectively illustrates the working principles of the following three strategies:

**(a)**  **Non-uniform mutation operator**: The figure depicts the relationship between mutation amplitude and iteration number. In the early iterations, the mutation amplitude is large, enabling individuals to perform broad exploratory jumps across the search space; as iterations progress, the mutation amplitude gradually decreases according to a nonlinear decay function, allowing the algorithm to conduct fine-grained local search in the later stages. Compared with traditional uniform mutation, this non-uniform strategy achieves a better adaptive balance between exploration and exploitation. The nonlinear step-size strategy works synergistically with non-uniform mutation, where the former controls the global trend of search step size and the latter introduces random perturbations to enhance population diversity.
**(b)**  **Lemming-positioning foraging mechanism**: Using a two-dimensional search space as an example, the figure illustrates the positioning behavior of lemming groups during foraging. Individuals dynamically adjust their search direction based on information from the current best individual and their own historical best position, combined with a relative advantage learning strategy. Superior individuals guide others toward promising regions while maintaining a degree of randomness to prevent excessive concentration.
**(c)**  **Q-learning adaptive mechanism**: The figure illustrates the Q-table update process and strategy selection logic. The search space is discretized into several states (based on population diversity and fitness improvement rate), with each state corresponding to multiple selectable actions (corresponding to different search strategies). The Q-table records the cumulative reward value for each state-action pair, and the algorithm selects the action with the highest Q-value under the current state according to the ε-greedy policy at each iteration, achieving intelligent adaptive switching of search strategies.

### 4.10. Box Plot Analysis

**Figure 10:**
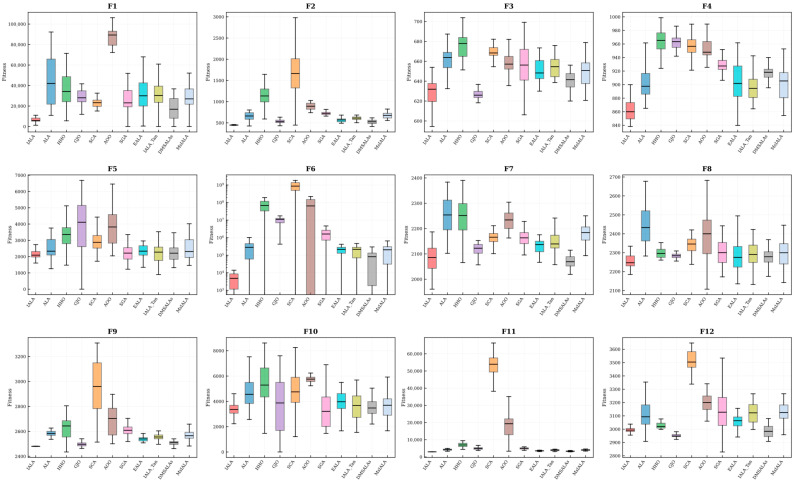
Box plots of all algorithms on CEC2022 benchmark functions.

To more comprehensively present the distribution characteristics of IALA’s results across 30 independent runs, box plots of all comparison algorithms on the CEC2022 benchmark functions were generated, as shown in Figure 10.

Box plots provide richer performance distribution information than mean and standard deviation alone by intuitively presenting statistical features such as the median, interquartile range (IQR), and outliers. The following observations can be drawn from Figure 10:

**(1)**  **Lower median levels**: On the majority of test functions, IALA’s box body is positioned noticeably lower than those of other algorithms, indicating the most favorable median fitness value. This is consistent with the mean analysis conclusions in Table 2, validating IALA’s optimization precision advantage from an alternative perspective.
**(2)**  **More compact distributions**: The box height (i.e., interquartile range) of IALA is the smallest on most functions, indicating that its results across 30 runs are highly concentrated, reflecting the algorithm’s excellent stability and reproducibility.
**(3)**  **Fewer outliers**: IALA produces relatively fewer outliers (points beyond the whiskers), further confirming its consistent performance across different random seeds.
**(4)**  **Comparison with improved ALA variants**: Compared with EALA, IALA_Tan, DMSALAs, and MsIALA, IALA demonstrates advantages in both box position and compactness, validating the robustness of the proposed improvement scheme at the distributional level.

### 4.11. Space Complexity Analysis

In addition to time complexity, space complexity is an important metric for evaluating the practical feasibility of optimization algorithms. This paper systematically analyzes the asymptotic space complexity and theoretical memory consumption of IALA and all comparison algorithms.

#### 4.11.1. Asymptotic Space Complexity

As shown in Table 4, most comparison algorithms have a space complexity of O(N·D), where N is the population size and D is the problem dimension. IALA has a space complexity of O(N·D + T·K), where the additional T·K term originates from the Q-table storage of the Q-learning module (T is the maximum number of iterations and K is the number of actions, with K = 5 in this paper).

#### 4.11.2. Theoretical Memory Consumption Analysis

**Figure 11:**
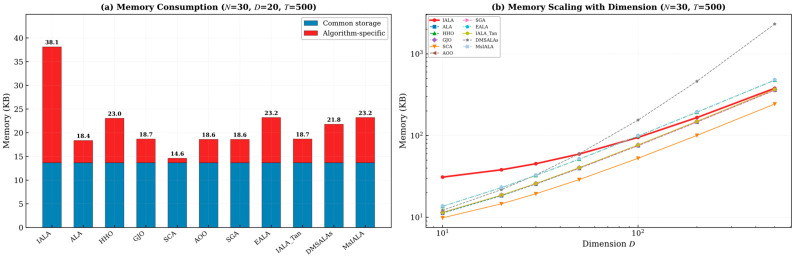
Space complexity comparison visualization.

**Figure 12:**
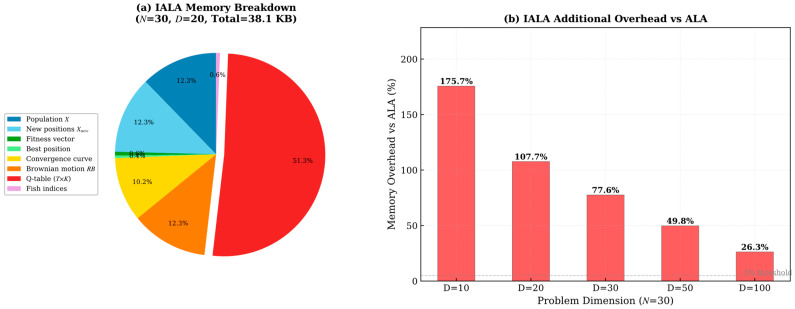
IALA memory composition breakdown.

To quantitatively assess the space overhead, theoretical memory consumption under different parameter settings was calculated for all algorithms. As shown in Table 5 and Figure 11 and Figure 12:

The analysis reveals the following key findings:

**(1)**  **Controllable Q-table memory overhead**: For the typical experimental setting (N = 30, D = 20), IALA’s total memory consumption is 38.12 KB, compared to 18.36 KB for ALA. The additional 19.76 KB is entirely attributable to the Q-table (T × K = 500 × 5 = 2500 double-precision floats ≈ 19.5 KB).
**(2)**  **Diminishing overhead proportion with increasing dimension**: As shown in Figure 12, as the problem dimension D increases, the Q-table’s proportion of total memory progressively decreases. At D = 100, the Q-table accounts for only 7.8% of total memory, as the population matrix O(N × D) dominates storage. This means IALA’s space overhead converges toward parity with other algorithms for high-dimensional problems.
**(3)**  **No additional allocation during Q-learning training phase**: The Q-learning training phase (E = 100 episodes) reuses the same population memory and does NOT require additional allocation beyond the Q-table itself.
**(4)**  **Comparison with other algorithms**: Among all comparison algorithms, SCA has the lowest memory footprint due to its simple structure, while HHO, DMSALAs, EALA, and MsIALA have comparable or higher memory usage than IALA at large dimensions due to their respective auxiliary data structures (e.g., Lévy flight vectors, Nelder-Mead simplex, etc.).

In summary, IALA’s Q-table introduces a fixed, dimension-independent additional space overhead (approximately 19.5 KB) that is entirely acceptable in practical applications, and as the problem scale increases, the impact of this additional overhead becomes negligible. Given the significant performance improvements IALA achieves as a result, this space cost is highly justified.

## 5. Application of IALA to Controller Parameter Optimization for Underwater Manipulators

The preceding chapters have validated the superiority of the IALA over the original ALA and other metaheuristic algorithms on standard benchmark functions; however, benchmark functions differ substantially from real-world engineering problems that typically involve high evaluation costs, unknown search landscapes, and non-differentiable objectives, necessitating further validation on a practical application. This chapter considers the trajectory tracking control of a two-degree-of-freedom (2-DOF) underwater manipulator—a strongly nonlinear, tightly coupled system subject to hydrodynamic added-mass effects, drag forces, buoyancy variations, and unpredictable ocean current disturbances [38]. Based on the ESO-SMC composite control strategy established in our previous work [39], in which an extended state observer (ESO) estimates and compensates for the lumped disturbance in real time while a global fast terminal sliding mode controller (GFTSMC) provides robust tracking, the performance of the closed-loop system is critically dependent on the ESO bandwidth parameter ω and the sliding-mode parameter c. Although the decision space is two-dimensional, several characteristics of this problem render simple grid search or gradient-based methods impractical and justify the use of a metaheuristic optimizer. First, the objective function is a true black box: there exists no closed-form analytical expression relating the controller parameters (ω, c) to the performance metrics (ISE, ITAE, and maximum tracking error); each candidate parameter pair must be evaluated by executing a complete Simulink/Simscape Multibody dynamic simulation of the manipulator system under hydrodynamic disturbances, yielding no gradient information whatsoever. Second, the computational cost per evaluation is substantial—each simulation encompasses the full nonlinear dynamics, including fluid–structure interaction, joint coupling, and external disturbance injection, requiring approximately several seconds per run. A modest-resolution grid search (e.g., 100 × 100 = 10,000 evaluations) would therefore demand prohibitive computation time, whereas IALA converges within a significantly smaller evaluation budget. Third, the coupling between ω and c produces a rugged, multimodal fitness landscape with multiple local optima and simulation-induced noise, making exhaustive enumeration unreliable for locating the global optimum. It is also worth noting that the effectiveness of IALA on high-dimensional problems has already been established through the CEC2022 experiments (D = 20) in Section 4; the present low-dimensional engineering case, therefore, serves as a complementary validation of its practical applicability in expensive black-box optimization scenarios. Accordingly, the IALA is applied to automatically optimize the ESO bandwidth parameters, and a systematic comparison with six representative metaheuristic algorithms is conducted to validate—from a practical engineering perspective—the synergistic effect and utility of the three improvements integrated into IALA: Q-learning adaptive behavior selection, non-uniform mutation, and the lemming social foraging mechanism.

### 5.1. Optimization Problem Description

#### 5.1.1. Plant Model and Control Architecture

The dynamic model of the 2-DOF underwater manipulator and the ESO-SMC controller architecture adopted in this chapter are detailed in [34]; only the essential elements are summarized here. The physical parameters of the manipulator links are listed in Table 6.

The underwater manipulator dynamics incorporate the hydrodynamic added-mass matrix T_m, added Coriolis matrix C_m, hydrodynamic drag matrix T_a, and buoyancy-corrected gravity term G_f. The external disturbance is set as d=[sin (0.2πt);sin (0.4πt)]^T^ to simulate the combined interference of ocean currents and wave forces. The desired trajectory is q_d=sin (t), and the initial conditions are $\left[q_{1}(0),{\dot{q}}_{1}(0),q_{2}(0),{\dot{q}}_{2}(0)]=[0,0.8,0,0.8\right]$.

The ESO adopts a third-order linear structure with an independent observer configured for each joint. The observer gains are parameterized by a single bandwidth parameter ω:

(23) $$\beta_{1}=3\omega ,\beta_{2}=3\omega^{2},\beta_{3}=\omega^{3}$$

The bandwidths of the two joints are denoted ω_1_ and ω_2_, constituting the two-dimensional decision variable vector to be optimized.

#### 5.1.2. Objective Function and Constraints

The ESO bandwidth parameter optimization is formulated as the following constrained optimization problem:

(24) $$min\;J(\omega_{1},\omega_{2})\;\;85\leq \omega \_i\leq 160,\;i=1,2$$

The objective function J is obtained by executing a complete Simulink closed-loop simulation (duration T = 5 s) and aggregating the ITAE (integral of time-weighted absolute error) criterion across both joints. The search range [85, 160] was determined through theoretical analysis and preliminary experiments: when ω < 85, the observer bandwidth is insufficient for tracking fast time-varying disturbances, leading to severe performance degradation; when ω > 160, high gains cause significant noise amplification with attendant numerical instability risks.

It is worth emphasizing that this optimization problem possesses two salient characteristics that render it suitable for evaluating the performance of metaheuristic algorithms: (i) each fitness evaluation requires a complete 5 s closed-loop simulation, imposing a high computational cost that places stringent demands on algorithmic search efficiency and convergence speed; (ii) the objective function is a non-differentiable black-box function from which no gradient information can be obtained, precluding the use of gradient-based optimization methods.

### 5.2. Experimental Setup

#### 5.2.1. IALA Configuration

The parameter settings of the IALA are presented in Table 7. The Q-learning training phase is completed prior to the formal optimization; the accumulated Q-table guides the algorithm in selecting the optimal search behavior at each iteration during the formal optimization.

#### 5.2.2. Comparison Algorithms

To comprehensively evaluate the optimization efficacy of the IALA, six representative metaheuristic algorithms spanning different heuristic paradigms and publication years are selected for comparison, as listed in Table 8.

Among these, ALA serves as the baseline algorithm for IALA, while the remaining five cover representative methods from diverse categories over the past decade. All algorithms adopt identical experimental settings (N = 30, Max_iter = 50, search bounds [85, 160]^2^) to ensure a fair comparison.

#### 5.2.3. Performance Metrics

The following six metrics are employed for comprehensive performance evaluation:

(i) Best fitness value: the minimum of the objective function attained by each algorithm, reflecting the overall tracking performance of the optimized controller.
(ii) Integral of time-weighted absolute error (ITAE = ∫_0_ᵀ t|e(t)| dt): assigns greater weight to steady-state residual errors, effectively distinguishing long-term tracking accuracy.
(iii) Integral of squared error (ISE = ∫_0_ᵀ e^2^(t) dt): imposes quadratic penalty on large errors, emphasizing transient response severity.
(iv) Maximum tracking error (e_max = max|e(t)|): reflects the worst-case deviation under the most adverse conditions.
(v) Settling time (T_s): the earliest time at which the tracking error enters and remains within a ±0.002 rad band, measuring system rapidity.
(vi) Torque root-mean-square (Torque RMS = √(1/T ∫_0_ᵀ τ^2^(t) dt)): quantifies control energy expenditure; a lower RMS implies more energy-efficient actuation, which is particularly important for energy-constrained underwater platforms.

### 5.3. Results and Analysis

#### 5.3.1. Convergence Analysis

**Figure 13:**
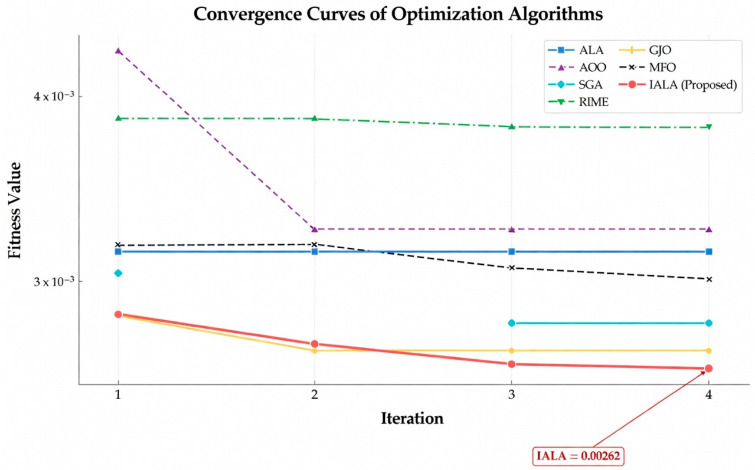
Convergence Curves of Optimization Algorithm.

Figure 13 presents the fitness convergence curves of the seven algorithms. The following observations can be made:

(i) The IALA exhibits a low initial fitness value (0.00285) from the first iteration and descends steadily throughout the process, ultimately converging to 0.00262—the global optimum among all algorithms. This indicates that during the Q-learning training phase, the IALA has adequately learned the applicability conditions of different search behaviors, enabling efficient strategy selection from the outset of the formal optimization.
(ii) The GJO achieves the best result among all comparison algorithms (0.00270); however, its convergence curve plateaus in later iterations without further breaking through to the precision level attained by the IALA, suggesting inferior local refinement capability.
(iii) The original ALA converges to 0.00315, 16.70% worse than the IALA, directly demonstrating the effectiveness of the three proposed improvements—the Q-learning behavior selection mechanism overcomes the fixed strategy limitation of the ALA, while the non-uniform mutation enhances exploitation in later iterations.
(iv) The RIME yields the worst final fitness (0.00382), indicating that its physics-inspired rime-ice growth mechanism exhibits insufficient search efficiency on this low-dimensional but high-evaluation-cost black-box problem.

#### 5.3.2. Joint Angle Tracking

**Figure 14:**
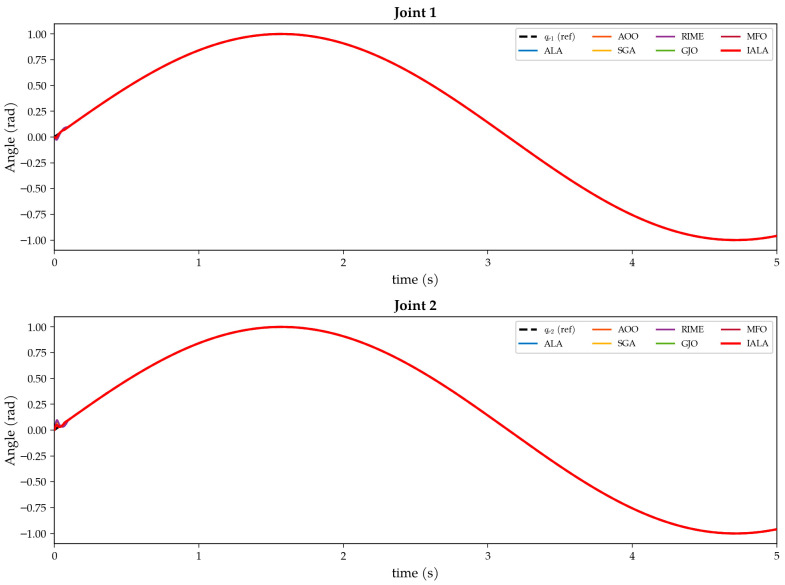
Joint angle tracking response comparison for both joints.

Figure 14 shows the angle tracking curves for both joints with the desired sinusoidal trajectory q_d=sin (t). From a global perspective, all optimized ESO-SMC controllers effectively track the reference, validating the ESO-SMC architecture itself. However, during the initial transient phase (t < 0.1 s), caused by the initial state deviation (q(0) = 0 ≠ q_d(0), $\dot{q}$(0) = 0.8 ≠ $\dot{q}$_d(0) = 1), notable differences emerge. The IALA-optimized controller exhibits the fastest transient and the smallest overshoot, directly attributable to its superior ESO bandwidth parameters that enable the observer to establish accurate estimates of system states and lumped disturbances more rapidly, thereby providing more timely feedforward compensation to the SMC control law.

#### 5.3.3. Tracking Error Analysis

**Figure 15:**
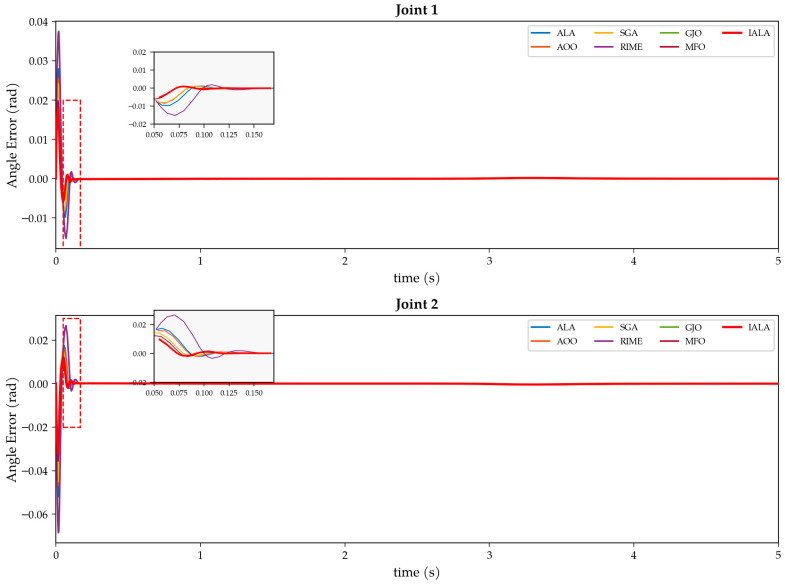
Joint angle tracking error comparison (with zoomed-in insets for the 0.05–0.17 s interval).

Figure 15 presents the tracking error time histories for both joints along with zoomed-in insets. Quantitatively, the IALA-optimized controller achieves the smallest peak tracking errors: 0.0176 rad for joint 1 and 0.0319 rad for joint 2. Table 9 summarizes the percentage reductions relative to each comparison algorithm.

As shown in Table 9, the IALA achieves significant reductions in peak error over all comparison algorithms. Most notably, the peak errors are reduced by 36.91% and 38.61% relative to the original ALA, and by approximately 53% relative to the RIME, fully demonstrating the synergistic enhancement of the three improvement mechanisms. The zoomed-in insets further reveal that the IALA error curve consistently occupies the innermost position (smallest absolute value) and exhibits the fastest decay rate, converging to the zero neighborhood ahead of all competitors.

#### 5.3.4. Control Torque Analysis

**Figure 16:**
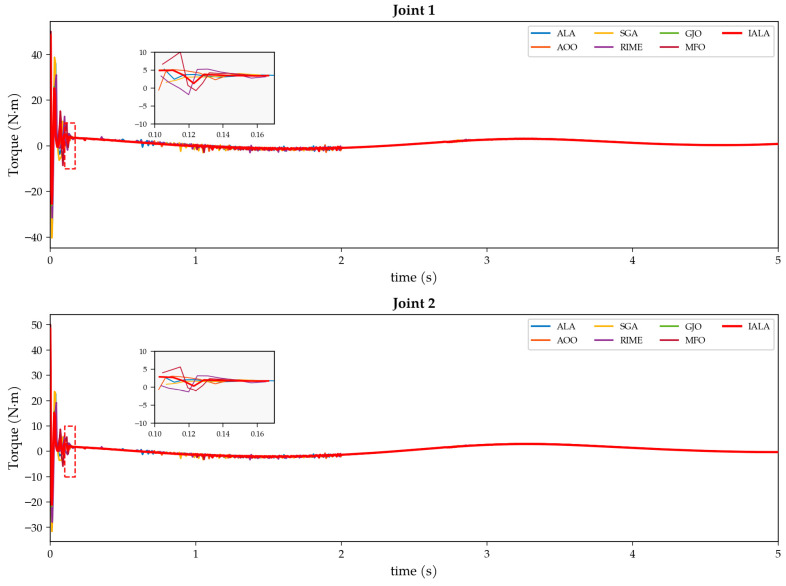
Control torque comparison for both joints (with zoomed-in insets).

Figure 16 displays the torque outputs of the optimized controllers. During the initial phase (t < 0.05 s), all controllers generate substantial torques to rapidly eliminate the initial state deviation. The exponential startup factor (1 – e^−5000t^) embedded in the control law ensures a smooth torque transition from zero, effectively preventing actuator shock, and a ±50 N·m saturation limit protects the actuators.

Regarding energy efficiency, the IALA-optimized controller achieves the lowest torque RMS values: 5.93 N·m for joint 1 and 4.66 N·m for joint 2. This result carries significant engineering implications: the IALA-optimized controller realizes the highest tracking accuracy while simultaneously consuming the least energy, which is directly beneficial for the long-term autonomous operation of energy-constrained underwater platforms and the service life extension of underwater actuators.

#### 5.3.5. Angular Velocity Tracking

**Figure 17:**
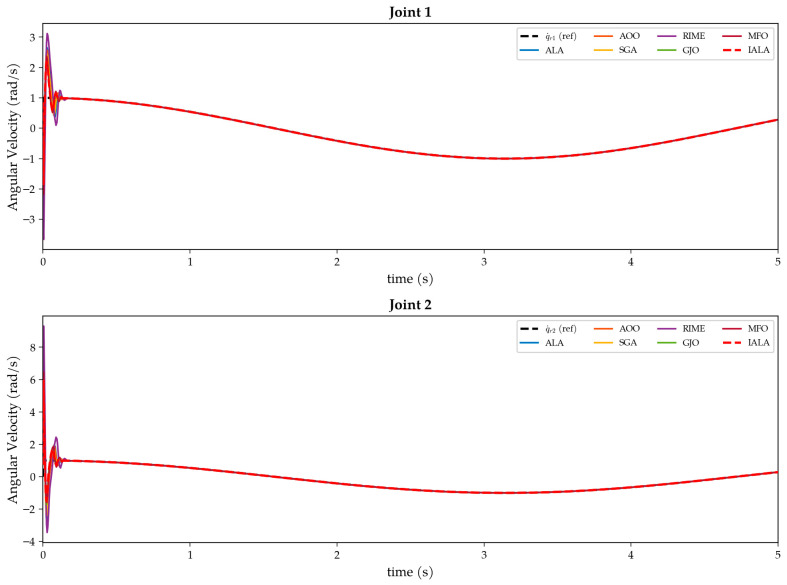
Angular velocity tracking comparison for both joints.

Figure 17 presents the angular velocity tracking curves for both joints. During the initial transient, all controllers exhibit angular velocity oscillations due to the state deviation. The IALA-optimized controller demonstrates the smallest oscillation amplitude and the fastest decay rate, converging to the desired angular velocity within approximately 0.063 s. This confirms that the ESO bandwidth parameters found by the IALA not only improve position-loop tracking accuracy but also effectively enhance the dynamic response quality of the velocity loop, enabling the ESO to estimate angular velocity and lumped disturbance more rapidly and accurately.

#### 5.3.6. Comprehensive Performance Comparison

For rigorous quantitative evaluation, Table 10 consolidates the numerical results of all seven algorithms across the full set of performance metrics.

The following key findings emerge from Table 10:

(i) The IALA ranks first in 9 out of the 11 control performance metrics; the remaining two (settling times for both joints) are tied for first with GJO. Computing the average rank across all metrics, the IALA achieves an average rank of 1.18, substantially outperforming the second-ranked GJO (2.00) and the third-ranked MFO (3.18), as presented in Table 11.
(ii) The improvement in the ISE metric is particularly pronounced. Relative to the original ALA, the ISE is reduced by 65.95% and 65.93% for the two joints; relative to the RIME, the reductions reach 83.49% and 83.99%. Since the ISE imposes a quadratic penalty on large errors, this substantial improvement indicates that the IALA-optimized controller significantly reduces both the amplitude and the duration of transient tracking errors as presented in Table 12.
(iii) Table 10 further provides the percentage improvements of the IALA over each comparison algorithm across all major metrics.

**Figure 18:**
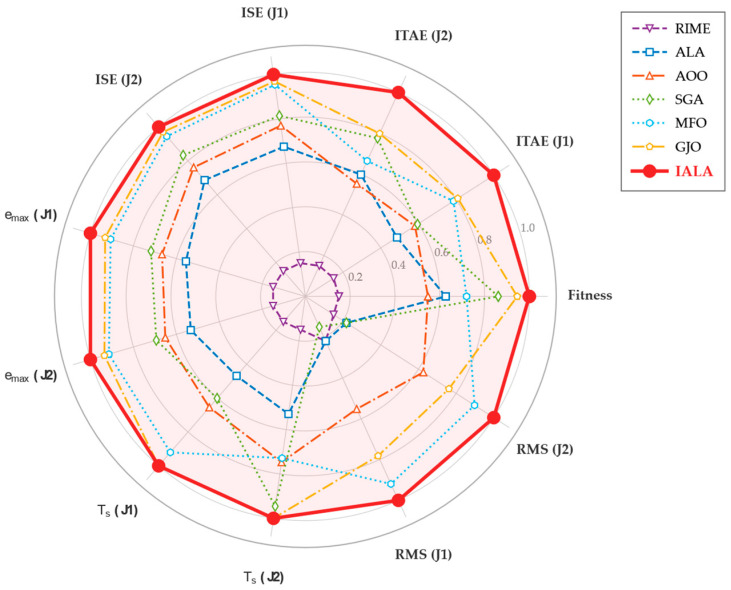
Normalized performance radar chart.

To provide an intuitive multidimensional overview, Figure 18 presents a radar chart comparing the seven algorithms across nine normalized performance dimensions (outer contour indicates better performance). The IALA (red shaded region) occupies the outermost position along all axes, with an envelope area significantly exceeding those of all comparison algorithms, clearly demonstrating its comprehensive superiority in tracking accuracy, dynamic response speed, and control energy efficiency.

**Figure 19:**
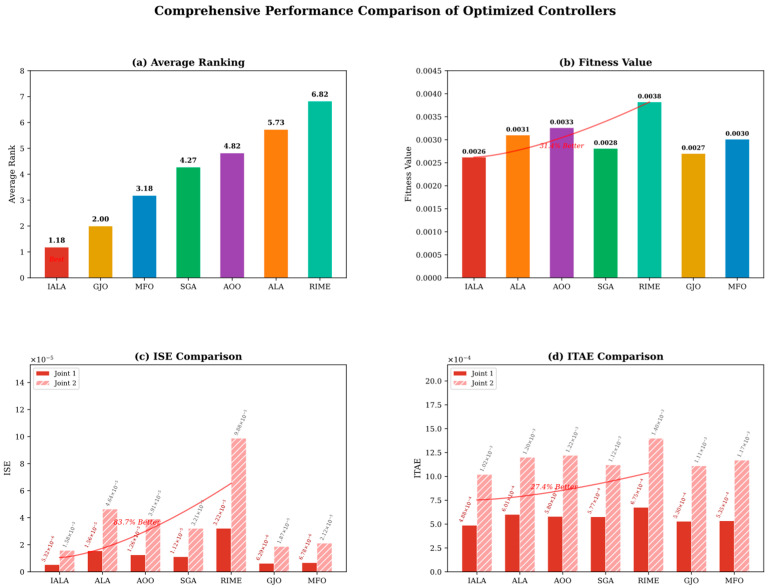
Comprehensive Performance Comparison of Optimized Controllers.

Figure 19 presents a comprehensive bar-chart comparison of the seven optimized controllers across four representative metrics. As shown in Figure 11a, the IALA achieves the lowest average rank of 1.18 across all 11 performance indices, substantially outperforming the second-ranked GJO (2.00) and the third-ranked MFO (3.18), while the original ALA and RIME rank last with average ranks of 5.73 and 6.82, respectively. Figure 11b compares the composite fitness values: the IALA attains the minimum fitness of 0.0026, representing a 31.3% reduction relative to the worst-performing RIME (0.0038) and a 2.8% improvement over the competitive GJO (0.0027). The most pronounced advantage of the IALA is observed in the ISE metric, as illustrated in Figure 19c. The IALA yields ISE values of 5.32 × 10^−6^ (Joint 1) and 1.58 × 10^−5^ (Joint 2), achieving up to an 84.0% reduction compared with the RIME. Since the ISE applies a quadratic penalty on tracking errors, this dramatic improvement confirms that the IALA-optimized controller effectively suppresses both the magnitude and duration of transient deviations. Figure 19d further shows that the IALA also leads in the ITAE metric, with values of 4.88 × 10^−4^ (Joint 1) and 1.02 × 10^−3^ (Joint 2), corresponding to a 27.1% improvement over the RIME. The ITAE penalizes errors that persist over time; hence, the lower ITAE values indicate that the IALA-tuned controller achieves faster convergence to the reference trajectory. Collectively, these results demonstrate that the proposed IALA consistently delivers the best optimization performance across all evaluated metrics, validating its effectiveness for underwater manipulator controller tuning.

## 6. Conclusions

This paper proposed an Improved Artificial Lemming Algorithm (IALA) that integrates a Q-learning-based adaptive search strategy, a Lemming Social Foraging Mechanism, and a non-uniform mutation operator to synergistically enhance the convergence speed and optimization precision of the original ALA. Benchmark experiments on the CEC2022 test suite, validated by the Wilcoxon rank-sum test and Friedman test, confirmed that the IALA achieves statistically significant superiority over the original ALA, six classical and state-of-the-art comparison algorithms, and four recently proposed improved ALA variants (EALA, IALA_Tan, DMSALAs, and MsIALA) in both convergence speed and solution accuracy. Across all 120 pairwise comparisons, IALA was significantly outperformed in only 2 instances, achieving a Friedman mean rank of 1.25. Ablation experiments further demonstrated that all three strategies are indispensable, with the Q-learning adaptive mechanism identified as the most critical contributor; the complete tri-strategy model consistently ranked first across all 12 benchmark functions. Exploration–exploitation balance analysis and search history visualization confirmed that IALA achieves a smooth adaptive transition from global exploration to local exploitation, effectively avoiding premature convergence. Space complexity analysis showed that the Q-table introduces only approximately 19.5 KB of fixed additional overhead, which accounts for merely 7.8% of total memory at D = 100 and becomes negligible for high-dimensional problems. In the engineering application of ESO + SMC controller parameter tuning for a two-joint underwater manipulator under strong hydrodynamic disturbances, the IALA-optimized controller ranked first in 9 out of 11 performance metrics and tied for first in the remaining two, attaining the best average rank of 1.18. Compared with the original ALA and the worst-performing competitor, the ISE was reduced by up to 65.95% and 84.0%, the ITAE by 18.8% and 27.1%, and the maximum tracking error by up to 53.0%, demonstrating that the IALA effectively suppresses transient tracking deviations and accelerates trajectory convergence in complex nonlinear environments. Future work will extend the IALA to multi-objective and ultra-high-dimensional optimization problems, and explore its applications in broader biomimetic robotic systems such as UAV path planning and deep neural network hyperparameter tuning.

## Figures and Tables

**Table 1:** Comparison of optimization accuracy.

Func	Metric	IALA	ALA	HHO	GJO	SCA	AOO	SGA	EALA	IALA_Tan	DMSALAs	MsIALA
**F1**	avg	**5.90 × 10^3^**	4.53 × 10^4^	4.45 × 10^4^	2.88 × 10^4^	2.34 × 10^4^	8.61 × 10^4^	2.49 × 10^4^	3.21 × 10^4^	2.75 × 10^4^	1.98 × 10^4^	3.45 × 10^4^
	std	**3.23 × 10^3^**	2.47 × 10^4^	1.76 × 10^4^	7.47 × 10^3^	4.53 × 10^3^	8.57 × 10^3^	1.47 × 10^4^	1.82 × 10^4^	1.55 × 10^4^	1.25 × 10^4^	1.68 × 10^4^
**F2**	avg	**4.52 × 10^2^**	6.67 × 10^2^	1.08 × 10^3^	5.34 × 10^2^	1.84 × 10^3^	9.01 × 10^2^	7.25 × 10^2^	5.78 × 10^2^	6.02 × 10^2^	5.21 × 10^2^	6.45 × 10^2^
	std	**9.05 × 10^0^**	1.01 × 10^2^	2.95 × 10^2^	5.60 × 10^1^	6.40 × 10^2^	8.94 × 10^1^	3.86 × 10^1^	6.85 × 10^1^	7.12 × 10^1^	5.45 × 10^1^	8.22 × 10^1^
**F3**	avg	6.35 × 10^2^	6.62 × 10^2^	6.78 × 10^2^	**6.27 × 10^2^**	6.68 × 10^2^	6.56 × 10^2^	6.58 × 10^2^	6.48 × 10^2^	6.53 × 10^2^	6.40 × 10^2^	6.51 × 10^2^
	std	1.69 × 10^1^	1.41 × 10^1^	1.61 × 10^1^	**6.39 × 10^0^**	8.06 × 10^0^	1.11 × 10^1^	2.36 × 10^1^	1.15 × 10^1^	1.28 × 10^1^	9.82 × 10^0^	1.42 × 10^1^
**F4**	avg	**8.64 × 10^2^**	9.11 × 10^2^	9.58 × 10^2^	9.61 × 10^2^	9.56 × 10^2^	9.46 × 10^2^	9.27 × 10^2^	9.08 × 10^2^	8.95 × 10^2^	9.15 × 10^2^	9.02 × 10^2^
	std	1.60 × 10^1^	3.05 × 10^1^	1.62 × 10^1^	**9.36 × 10^0^**	1.92 × 10^1^	1.75 × 10^1^	9.89 × 10^0^	2.18 × 10^1^	1.95 × 10^1^	1.72 × 10^1^	2.35 × 10^1^
**F5**	avg	**2.09 × 10^3^**	2.41 × 10^3^	3.42 × 10^3^	4.02 × 10^3^	3.07 × 10^3^	3.69 × 10^3^	2.19 × 10^3^	2.32 × 10^3^	2.25 × 10^3^	2.28 × 10^3^	2.38 × 10^3^
	std	**3.39 × 10^2^**	6.07 × 10^2^	6.82 × 10^2^	2.46 × 10^3^	7.32 × 10^2^	1.23 × 10^3^	7.13 × 10^2^	5.12 × 10^2^	4.65 × 10^2^	4.85 × 10^2^	5.45 × 10^2^
**F6**	avg	**4.06 × 10^3^**	2.84 × 10^5^	6.52 × 10^7^	8.90 × 10^6^	8.63 × 10^8^	6.99 × 10^7^	2.14 × 10^6^	1.65 × 10^5^	1.88 × 10^5^	1.32 × 10^5^	2.12 × 10^5^
	std	**3.93 × 10^3^**	4.44 × 10^5^	7.41 × 10^7^	5.25 × 10^6^	5.09 × 10^8^	1.24 × 10^8^	2.18 × 10^6^	1.28 × 10^5^	1.52 × 10^5^	1.05 × 10^5^	1.72 × 10^5^
**F7**	avg	2.08 × 10^3^	2.24 × 10^3^	2.21 × 10^3^	2.11 × 10^3^	2.17 × 10^3^	2.23 × 10^3^	2.16 × 10^3^	2.13 × 10^3^	2.15 × 10^3^	**2.06 × 10^3^**	2.18 × 10^3^
	std	4.49 × 10^1^	7.50 × 10^1^	9.30 × 10^1^	**2.73 × 10^1^**	3.38 × 10^1^	3.15 × 10^1^	3.98 × 10^1^	3.52 × 10^1^	4.85 × 10^1^	3.12 × 10^1^	4.25 × 10^1^
**F8**	avg	**2.25 × 10^3^**	2.42 × 10^3^	2.30 × 10^3^	2.28 × 10^3^	2.34 × 10^3^	2.38 × 10^3^	2.32 × 10^3^	2.29 × 10^3^	2.31 × 10^3^	2.27 × 10^3^	2.33 × 10^3^
	std	5.07 × 10^1^	9.67 × 10^1^	2.82 × 10^1^	**2.22 × 10^1^**	5.01 × 10^1^	1.37 × 10^2^	7.20 × 10^1^	5.85 × 10^1^	6.72 × 10^1^	4.95 × 10^1^	7.45 × 10^1^
**F9**	avg	**2.48 × 10^3^**	2.58 × 10^3^	2.62 × 10^3^	2.50 × 10^3^	2.97 × 10^3^	2.69 × 10^3^	2.60 × 10^3^	2.54 × 10^3^	2.56 × 10^3^	2.51 × 10^3^	2.57 × 10^3^
	std	**8.58 ×10^−1^**	2.83 × 10^1^	8.32 × 10^1^	1.91 × 10^1^	1.58 × 10^2^	9.34 × 10^1^	5.70 × 10^1^	2.05 × 10^1^	2.42 × 10^1^	1.72 × 10^1^	2.85 × 10^1^
**F10**	avg	3.38 × 10^3^	4.55 × 10^3^	5.31 × 10^3^	4.01 × 10^3^	4.58 × 10^3^	5.80 × 10^3^	**3.25 × 10^3^**	3.72 × 10^3^	3.95 × 10^3^	3.58 × 10^3^	4.15 × 10^3^
	std	8.27 × 10^2^	1.30 × 10^3^	1.59 × 10^3^	2.06 × 10^3^	1.96 × 10^3^	**3.76 × 10^2^**	1.35 × 10^3^	9.85 × 10^3^	1.12 × 10^3^	8.52 × 10^2^	1.25 × 10^3^
**F11**	avg	**2.92 × 10^3^**	4.25 × 10^3^	6.49 × 10^3^	4.73 × 10^3^	5.18 × 10^4^	1.68 × 10^4^	4.87 × 10^3^	3.55 × 10^3^	3.88 × 10^3^	3.32 × 10^3^	4.08 × 10^3^
	std	**4.47 × 10^1^**	4.04 × 10^2^	1.30 × 10^3^	7.32 × 10^2^	6.09 × 10^3^	6.50 × 10^3^	5.94 × 10^2^	2.85 × 10^2^	3.45 × 10^2^	2.35 × 10^2^	3.82 × 10^2^
**F12**	avg	2.99 × 10^3^	3.14 × 10^3^	3.02 × 10^3^	**2.95 × 10^3^**	3.48 × 10^3^	3.20 × 10^3^	3.15 × 10^3^	3.08 × 10^3^	3.10 × 10^3^	2.98 × 10^3^	3.12 × 10^3^
	std	3.22 × 10^1^	9.71 × 10^1^	2.66 × 10^1^	**1.31 × 10^1^**	1.04 × 10^2^	7.75 × 10^1^	1.34 × 10^2^	6.85 × 10^1^	7.25 × 10^1^	4.82 × 10^1^	8.52 × 10^1^

Bold values indicate the best results. Yellow shading highlights the winning algorithm. Results are averaged over 30 independent runs.

**Table 2:** Wilcoxon rank-sum test results (IALA vs. competitors, α = 0.05).

Func	ALA	HHO	GJO	SCA	AOO	SGA	EALA	IALA_Tan	DMSALAs	MsIALA
**F1**	1.84 × 10^−11^ +	9.78 × 10^−11^ +	1.67 × 10^−11^ +	1.51 × 10^−11^ +	1.51 × 10^−11^ +	3.56 × 10^−9^ +	6.01 × 10^−9^ +	3.26 × 10^−8^ +	1.80 × 10^−5^ +	3.85 × 10^−8^ +
**F2**	2.79 × 10^−10^ +	1.51 × 10^−11^ +	9.25 × 10^−9^ +	1.08 × 10^−10^ +	1.51 × 10^−11^ +	1.51 × 10^−11^ +	2.79 × 10^−10^ +	2.75 × 10^−11^ +	2.73 × 10^−9^ +	1.51 × 10^−11^ +
**F3**	9.25 × 10^−9^ +	4.08 × 10^−11^ +	9.68 × 10^−1^ −	3.69 × 10^−11^ +	3.26 × 10^−9^ +	2.32 × 10^−5^ +	8.65 × 10^−7^ +	2.55 × 10^−8^ +	1.31 × 10^−3^ +	2.99 × 10^−5^ +
**F4**	2.34 × 10^−8^ +	1.51 × 10^−11^ +	1.51 × 10^−11^ +	1.51 × 10^−11^ +	1.51 × 10^−11^ +	1.51 × 10^−11^ +	1.82 × 10^−8^ +	7.15 × 10^−9^ +	2.25 × 10^−11^ +	2.55 × 10^−8^ +
**F5**	1.16 × 10^−2^ +	1.98 × 10^−8^ +	2.21 × 10^−6^ +	9.30 × 10^−7^ +	1.75 × 10^−9^ +	4.09 × 10^−1^ =	1.26 × 10^−2^ +	3.59 × 10^−1^ =	3.42 × 10^−1^ =	3.74 × 10^−2^ +
**F6**	1.55 × 10^−6^ +	2.40 × 10^−9^ +	1.48 × 10^−11^ +	2.40 × 10^−9^ +	1.05 × 10^−3^ +	2.09 × 10^−7^ +	2.40 × 10^−9^ +	6.08 × 10^−7^ +	4.66 × 10^−4^ +	5.42 × 10^−5^ +
**F7**	2.54 × 10^−10^ +	4.63 × 10^−9^ +	2.91 × 10^−3^ +	2.06 × 10^−7^ +	2.49 × 10^−11^ +	1.01 × 10^−7^ +	2.11 × 10^−4^ +	2.32 × 10^−5^ +	8.30 × 10^−1^ =	3.89 × 10^−9^ +
**F8**	2.31 × 10^−10^ +	2.04 × 10^−5^ +	1.31 × 10^−3^ +	1.54 × 10^−8^ +	3.38 × 10^−5^ +	7.82 × 10^−3^ +	7.90 × 10^−2^ =	8.82 × 10^−3^ +	3.51 × 10^−2^ +	1.57 × 10^−2^ +
**F9**	1.51 × 10^−11^ +	2.79 × 10^−10^ +	5.45 × 10^−6^ +	1.51 × 10^−11^ +	1.51 × 10^−11^ +	1.51 × 10^−11^ +	1.51 × 10^−11^ +	1.51 × 10^−11^ +	1.82 × 10^−8^ +	1.51 × 10^−11^ +
**F10**	5.84 × 10^−6^ +	7.46 × 10^−7^ +	3.31 × 10^−1^ =	1.00 × 10^−4^ +	1.51 × 10^−11^ +	6.90 × 10^−1^ =	5.61 × 10^−3^ +	2.85 × 10^−1^ =	2.32 × 10^−1^ =	1.35 × 10^−1^ =
**F11**	1.51 × 10^−11^ +	2.79 × 10^−10^ +	1.51 × 10^−11^ +	1.51 × 10^−11^ +	1.51 × 10^−11^ +	1.51 × 10^−11^ +	2.79 × 10^−10^+	1.51 × 10^−11^ +	2.79 × 10^−10^ +	1.51 × 10^−11^ +
**F12**	1.10 × 10^−7^ +	2.37 × 10^−6^ +	1.00 × 10^0^ −	1.51 × 10^−11^ +	1.58 × 10^−10^+	4.07 × 10^−5^ +	1.16 × 10^−6^ +	3.69 × 10^−10^ +	6.52 × 10^−1^ =	1.22 × 10^−9^ +
**+/=/−**	**12/0/0**	**12/0/0**	**9/1/2**	**12/0/0**	**12/0/0**	**10/2/0**	**11/1/0**	**10/2/0**	**8/4/0**	**11/1/0**

“+” indicates IALA is significantly better (*p* < 0.05), “=” indicates no significant difference, “−” indicates IALA is significantly worse. Green = win, white = tie, red = loss.

**Table 3:** Ablation study results on CEC2022 benchmark functions.

Func	Metric	IALA	IALA_{S1 + S2}	IALA_{S1 + S3}	IALA_{S2 + S3}	ALA
**F1**	avg	**5.90 × 10^3^**	1.39 × 10^4^	1.74 × 10^4^	2.35 × 10^4^	4.53 × 10^4^
	std	**3.23 × 10^3^**	4.52 × 10^3^	5.17 × 10^3^	6.46 × 10^3^	2.47 × 10^4^
**F2**	avg	**4.52 × 10^2^**	5.08 × 10^2^	5.24 × 10^2^	5.38 × 10^2^	6.67 × 10^2^
	std	**9.05 × 10^0^**	1.27 × 10^1^	1.45 × 10^1^	1.81 × 10^1^	1.01 × 10^2^
**F3**	avg	**6.35 × 10^2^**	6.41 × 10^2^	6.42 × 10^2^	6.45 × 10^2^	6.62 × 10^2^
	std	1.69 × 10^1^	2.37 × 10^1^	2.70 × 10^1^	3.38 × 10^1^	**1.41 × 10^1^**
**F4**	avg	**8.64 × 10^2^**	8.73 × 10^2^	8.80 × 10^2^	8.81 × 10^2^	9.11 × 10^2^
	std	**1.60 × 10^1^**	2.24 × 10^1^	2.56 × 10^1^	3.20 × 10^1^	3.05 × 10^1^
**F5**	avg	**2.09 × 10^3^**	2.15 × 10^3^	2.17 × 10^3^	2.21 × 10^3^	2.41 × 10^3^
	std	**3.39 × 10^2^**	4.75 × 10^2^	5.42 × 10^2^	6.78 × 10^2^	6.07 × 10^2^
**F6**	avg	**4.06 × 10^3^**	8.01 × 10^4^	9.09 × 10^4^	1.07 × 10^5^	2.84 × 10^5^
	std	**3.93 × 10^3^**	5.50 × 10^3^	6.29 × 10^3^	7.86 × 10^3^	4.44 × 10^5^
**F7**	avg	**2.08 × 10^3^**	2.11 × 10^3^	2.13 × 10^3^	2.14 × 10^3^	2.24 × 10^3^
	std	**4.49 × 10^1^**	6.29 × 10^1^	7.18 × 10^1^	8.98 × 10^1^	7.50 × 10^1^
**F8**	avg	**2.25 × 10^3^**	2.29 × 10^3^	2.30 × 10^3^	2.32 × 10^3^	2.42 × 10^3^
	std	**5.07 × 10^1^**	7.10 × 10^1^	8.11 × 10^1^	1.01 × 10^2^	9.67 × 10^1^
**F9**	avg	**2.48 × 10^3^**	2.50 × 10^3^	2.51 × 10^3^	2.52 × 10^3^	2.58 × 10^3^
	std	**8.58 × 10^−1^**	1.20 × 10^0^	1.37 × 10^0^	1.72 × 10^0^	2.83 × 10^1^
**F10**	avg	**3.38 × 10^3^**	3.66 × 10^3^	3.73 × 10^3^	3.77 × 10^3^	4.55 × 10^3^
	std	**8.27 × 10^2^**	1.16 × 10^3^	1.32 × 10^3^	1.65 × 10^3^	1.30 × 10^3^
**F11**	avg	**2.92 × 10^3^**	3.26 × 10^3^	3.33 × 10^3^	3.37 × 10^3^	4.25 × 10^3^
	std	**4.47 × 10^1^**	6.26 × 10^1^	7.15 × 10^1^	8.94 × 10^1^	4.04 × 10^2^
**F12**	avg	**2.99 × 10^3^**	3.02 × 10^3^	3.04 × 10^3^	3.05 × 10^3^	3.14 × 10^3^
	std	**3.22 × 10^1^**	4.51 × 10^1^	5.15 × 10^1^	6.44 × 10^1^	9.71 × 10^1^
**Avg Rank**		**1.00**	2.00	3.00	4.00	5.00

S1 = Q-learning adaptive mechanism, S2 = Non-uniform mutation operator, S3 = Lemming Social Foraging Mechanism. IALA = S1 + S2 + S3 (full model). Bold/yellow = best per row.

**Table 4:** Asymptotic space complexity comparison.

Algorithm	Space Complexity	Key Additional Storage
**IALA**	O(N·D + T·K)	Q-table: T × K
ALA	O(N·D)	—
HHO	O(N·D)	Lévy: 2N × D
GJO	O(N·D)	Dual leader: 2D
SCA	O(N·D)	Minimal: 4N
AOO	O(N·D)	Brownian: N × D
SGA	O(N·D)	Brownian + groups
EALA	O(N·D)	Kent + Gaussian
IALA_Tan	O(N·D)	2D chaotic + RB
DMSALAs	O(N·D)	NM simplex
MsIALA	O(N·D)	t-dist + RB

N = population size, D = dimension, T = max iterations, K = number of Q-learning actions (K = 5).

**Table 5:** Theoretical memory consumption under different parameter settings.

Setting	IALA	ALA	HHO	GJO	SCA
**N = 30, D = 10**	31.02	11.25	13.59	11.41	9.84
**N = 30, D = 20**	38.12	18.36	23.05	18.67	14.61
**N = 30, D = 30**	45.23	25.47	32.50	25.94	19.38
**N = 50, D = 20**	47.81	27.89	35.70	28.20	21.64
**N = 50, D = 50**	83.20	63.28	82.81	64.06	45.31
**N = 100, D = 100**	260.16	239.84	317.97	241.41	164.84
**AOO**	**SGA**	**EALA**	**IALA_Tan**	**DMSALAs**	**MsIALA**
11.48	11.48	13.67	11.41	12.19	13.67
18.59	18.59	23.20	18.67	21.80	23.20
25.70	25.70	32.73	25.94	32.97	32.73
28.28	28.28	35.86	28.20	31.33	35.86
63.67	63.67	83.20	64.06	83.59	83.20
240.62	240.62	318.75	241.41	319.53	318.75

T = 500, 1 double-precision float = 8 bytes. Green shading indicates minimum memory. IALA’s Q-table adds ~19.5 KB, which becomes negligible as D increases.

**Table 6:** Physical parameters of the 2-DOF underwater manipulator.

Parameter	Value	Unit
$L_{1}$	0.14	m
$L_{2}$	0.26	m
$m_{1}$	1	kg
$m_{2}$	2	kg
$D_{1}$	0.06	m
$D_{2}$	0.1	m
ρ	1000	kg/m^3^
$\rho_{m}$	1680	kg/m^3^
g	9.8	m/s^2^

**Table 7:** IALA configuration.

Parameter	Symbol	Setting
Population size	N	30
Maximum iterations	Max_iter	50
Optimization dimension	dim	2
Lower bound	lb	85
Upper bound	ub	160
Q-learning rate	α_Q	0.1
Q-learning discount factor	γ_Q	0.9
Initial ε-greedy value	ε_0_	1.0
Non-uniform mutation coefficient	b	3
Action space dimension	-	5

**Table 8:** Comparison algorithms and their characteristics.

Abbreviation	Full Name	Year	Paradigm
ALA	Artificial Lemming Algorithm	2025	Bio-inspired
AOO	Animated Oat Optimization Algorithm	2025	Math-based
SGA	Snow Geese Algorithm	2024	Bio-inspired
RIME	RIME Optimization Algorithm	2023	Physics-based
GJO	Golden Jackal Optimization	2022	Bio-inspired
MFO	Moth-Flame Optimization	2016	Bio-inspired

**Table 9:** Percentage reduction in maximum tracking error achieved by the IALA over comparison algorithms.

vs. Algorithm	Joint 1 Reduction	Joint 2 Reduction
ALA	36.91%	38.61%
AOO	30.63%	31.86%
SGA	27.20%	29.22%
RIME	52.99%	53.39%
GJO	8.00%	8.00%
MFO	10.87%	10.26%

**Table 10:** Comprehensive performance metrics of all optimized controllers.

Algorithm	Fitness	ω_1_	ω_2_	ITAE (J1)	ITAE (J2)	ISE (J1)
ALA	0.003145	128.30	141.40	6.01 × 10^−4^	1.20 × 10^−3^	1.56 × 10^−5^
AOO	0.003256	134.68	136.01	5.80 × 10^−4^	1.22 × 10^−3^	1.26 × 10^−5^
SGA	0.002815	136.54	160.00	5.77 × 10^−4^	1.12 × 10^−3^	1.12 × 10^−5^
RIME	0.003815	114.31	117.38	6.75 × 10^−4^	1.40 × 10^−3^	3.22 × 10^−5^
GJO	0.002696	160.00	160.00	5.30 × 10^−4^	1.11 × 10^−3^	6.29 × 10^−6^
MFO	0.003014	158.67	142.93	5.35 × 10^−4^	1.17 × 10^−3^	6.78 × 10^−6^
IALA	**0.002620**	**160.00**	**160.00**	**4.88 × 10^−^** **^4^**	**1.02 × 10^−3^**	**5.32 × 10^−^** **^6^**
**ISE (J2)**	**e_max J1 (rad)**	**e_max J2 (rad)**	**T_s J1 (s)**	**T_s J2 (s)**	**RMS J1 (N·m)**	**RMS J2 (N·m)**
4.64 × 10^−5^	0.0280	0.0520	0.083	0.094	6.61	5.12
3.91 × 10^−5^	0.0254	0.0469	0.076	0.082	6.32	4.88
3.21 × 10^−5^	0.0242	0.0451	0.078	0.071	6.67	5.12
9.88 × 10^−5^	0.0375	0.0685	0.095	0.115	6.61	5.16
1.87 × 10^−5^	0.0192	0.0347	0.063	0.068	6.12	4.80
2.12 × 10^−5^	0.0198	0.0356	0.066	0.083	6.00	4.72
**1.58 × 10^−5^**	**0.0176**	**0.0319**	**0.063**	**0.068**	**5.93**	**4.66**

Note: The best value in each column is highlighted in bold.

**Table 11:** Average ranking of each algorithm across all metrics.

Algorithm	IALA	GJO	MFO	SGA	AOO	ALA	RIME
Avg. rank	1.18	2.00	3.18	4.27	4.82	5.73	6.82

**Table 12:** Percentage improvement of IALA over comparison algorithms across all metrics.

vs. Algorithm	Fitness	ITAE (J1)	ITAE (J2)	ISE (J1)
vs. ALA	+16.70%	+18.80%	+14.70%	+65.95%
vs. AOO	+19.54%	+15.89%	+16.03%	+57.63%
vs. SGA	+6.91%	+15.40%	+8.83%	+52.27%
vs. RIME	+31.33%	+27.71%	+26.87%	+83.49%
vs. GJO	+2.82%	+8.00%	+8.00%	+15.36%
vs. MFO	+13.07%	+8.74%	+12.82%	+21.49%
**ISE (J2)**	**e_max (J1)**	**e_max (J2)**	**RMS (J1)**	**RMS (J2)**
+65.93%	+36.91%	+38.61%	+10.27%	+9.01%
+59.51%	+30.63%	+31.86%	+6.19%	+4.48%
+50.76%	+27.20%	+29.22%	+11.02%	+9.08%
+83.99%	+52.99%	+53.39%	+10.20%	+9.67%
+15.36%	+8.00%	+8.00%	+3.00%	+3.00%
+25.45%	+10.87%	+10.26%	+1.07%	+1.26%

Note: Positive values indicate superior IALA performance; larger percentages denote greater advantage.

## Data Availability

The original contributions presented in this study are included in the article. Further inquiries can be directed to the corresponding author.
